# Supplementary material for: Polyaromatic nanocapsules as photoresponsive hosts in water
Source: Nat Commun. 2019 Apr 24;10:1948. doi: 10.1038/s41467-019-09928-x (PMC6482177; doi:10.1038/s41467-019-09928-x)
Supplement: Supplementary file 1 — Supplementary Information [file 41467_2019_9928_MOESM1_ESM.pdf]

Supplementary Information

Polyaromatic nanocapsules as photoresponsive hosts in water

Catti et al.

Supplementary Figures

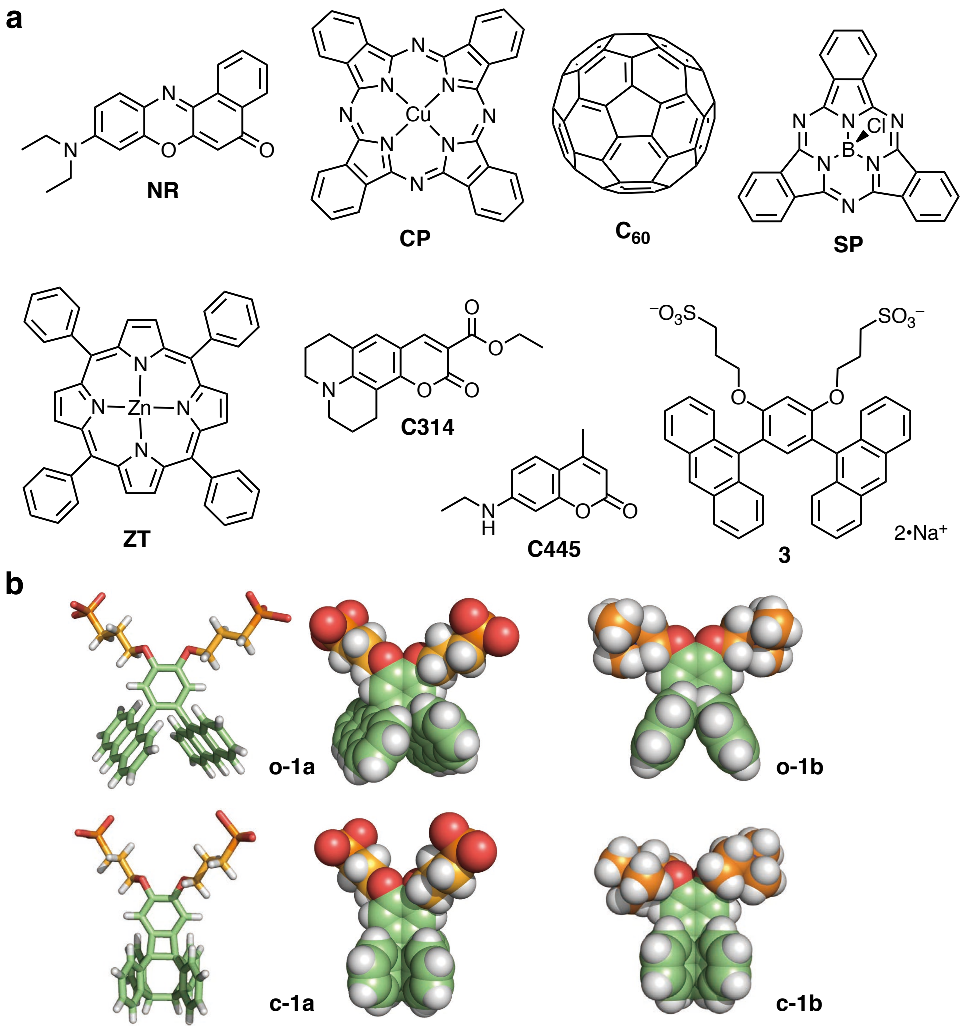

Supplementary Fig. 1 (a) Chemical structures of guest molecules and V-shaped amphiphile **3** and (b) optimized structures (DFT, B3LYP/6-31G (d) level) of open and closed V-shaped amphiphiles.

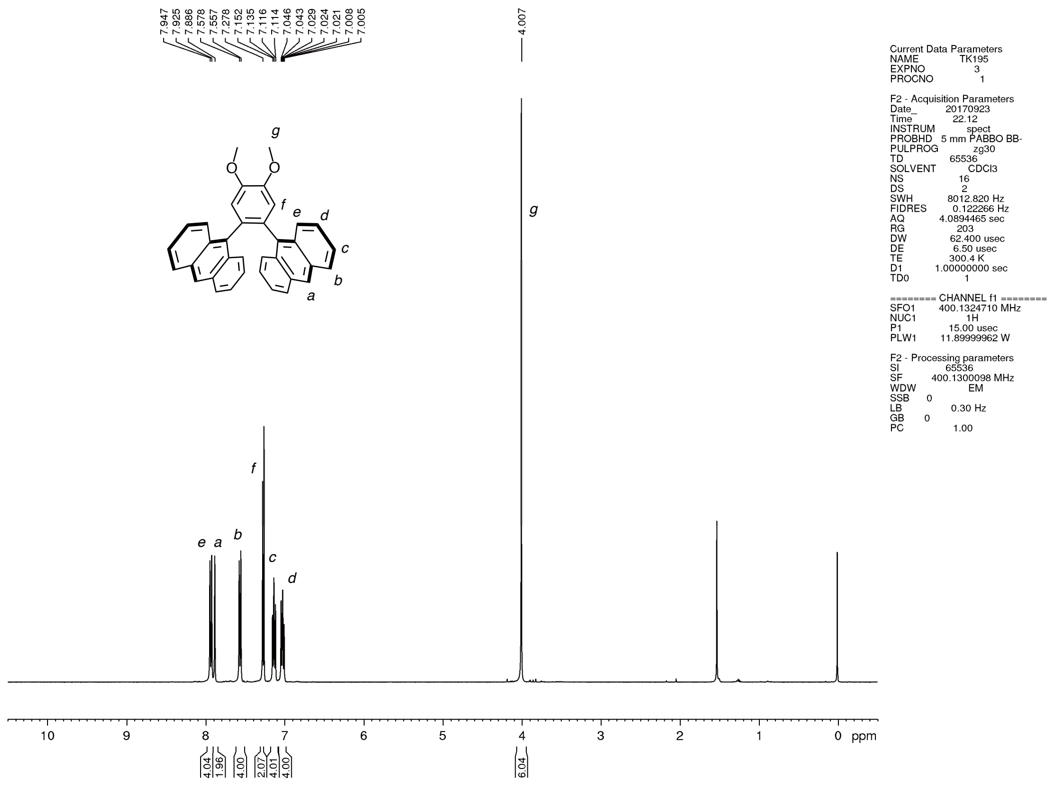

Supplementary Fig. 2 ^{1}H NMR spectrum (400 MHz, $CDCl_3$, room temperature) of $\mathbf{1_{OMe}}$.

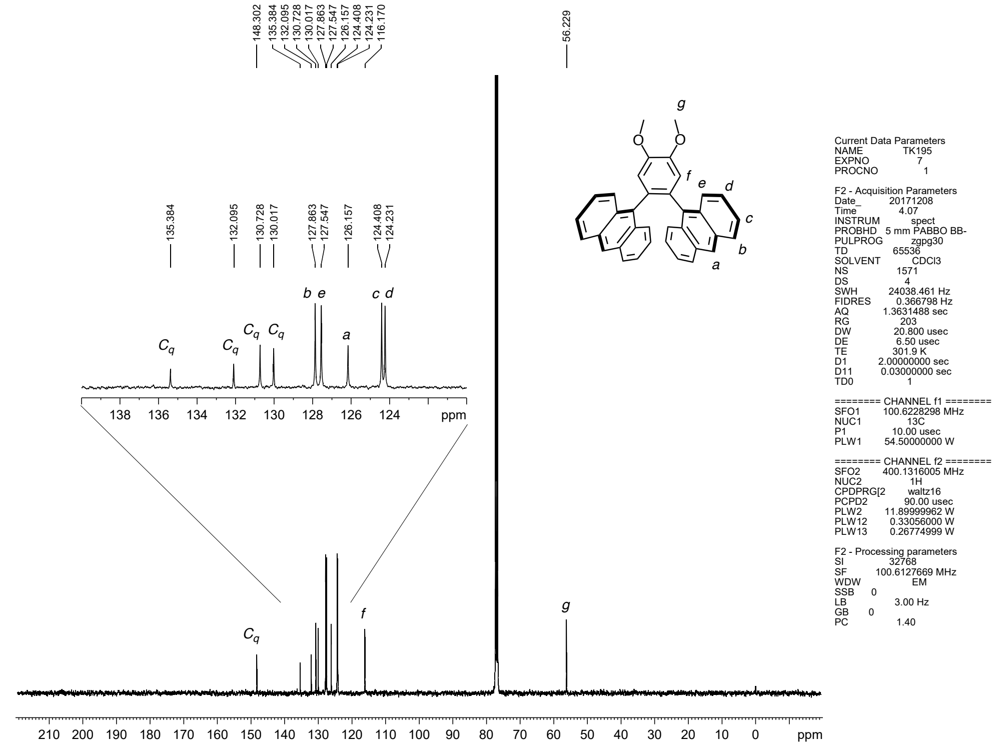

Supplementary Fig. 3 ^{13}C NMR spectrum (100 MHz, $CDCl_3$, room temperature) of $\mathbf{1_{OMe}}$.

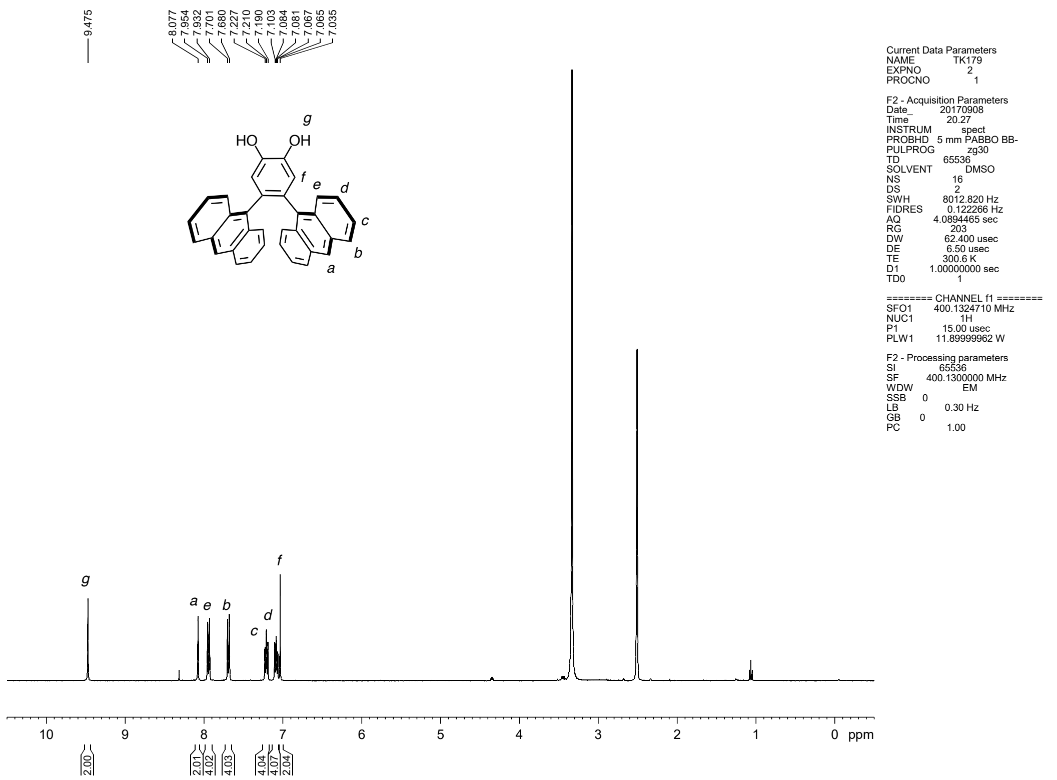

Supplementary Fig. 4 ^{1}H NMR spectrum (400 MHz, DMSO-d_6, room temperature) of $\mathbf{1_{OH}}$.

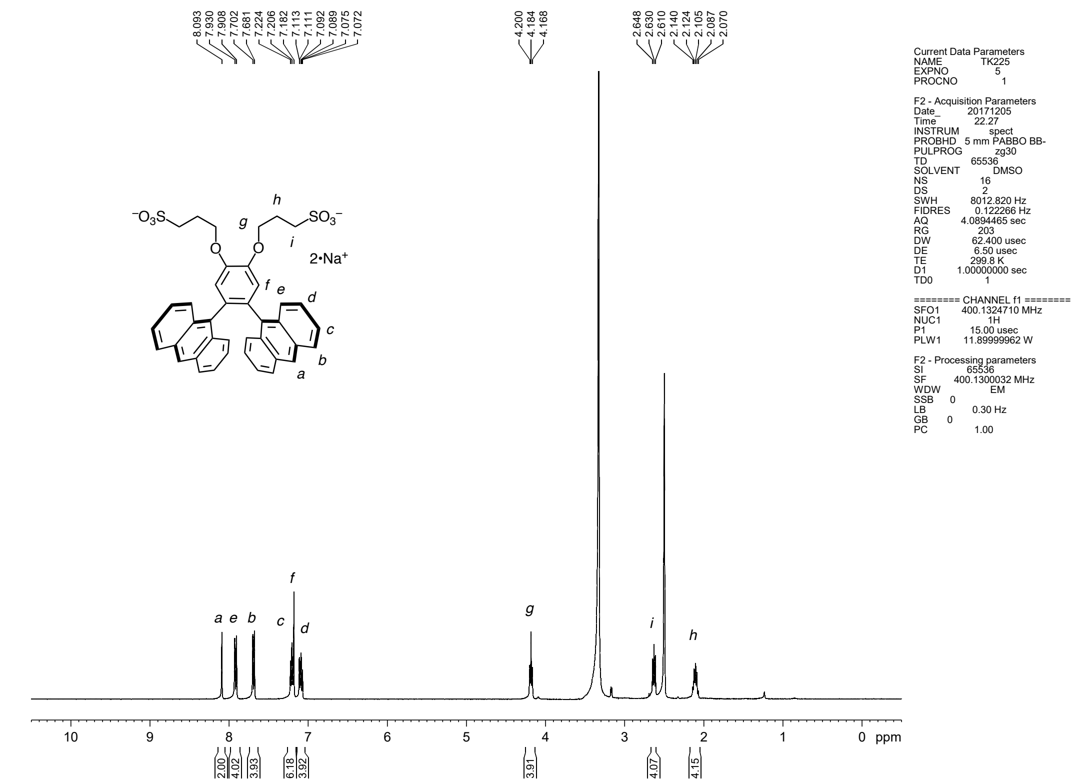

Supplementary Fig. 5 ^{1}H NMR spectrum (400 MHz, DMSO-d_6, room temperature) of **o-1a**.

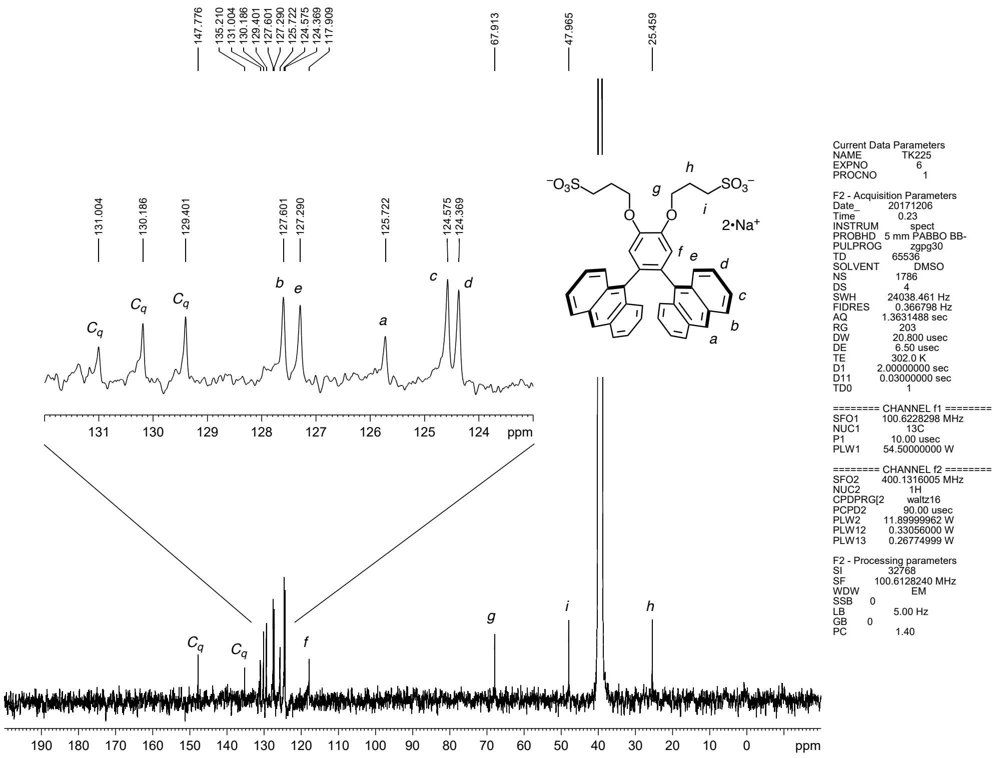

Supplementary Fig. 6 ^{13}C NMR spectrum (100 MHz, DMSO-d_6, room temperature) of **o-1a**.

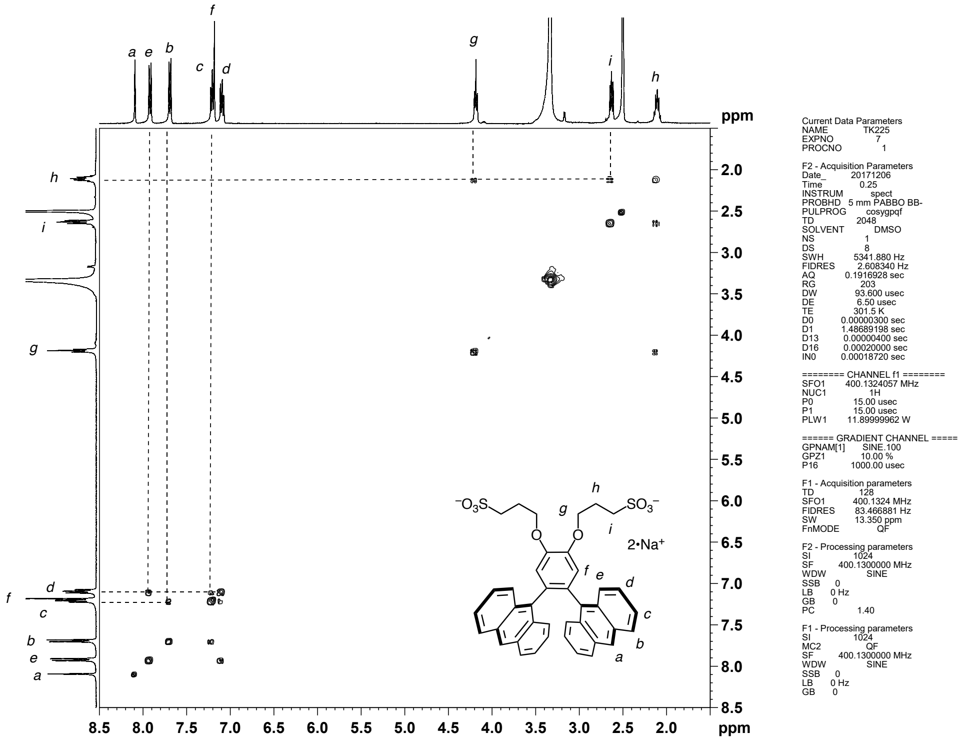

Supplementary Fig. 7 ^{1}H-^{1}H COSY spectrum (400 MHz, DMSO-d_6, room temperature) of **o-1a**.

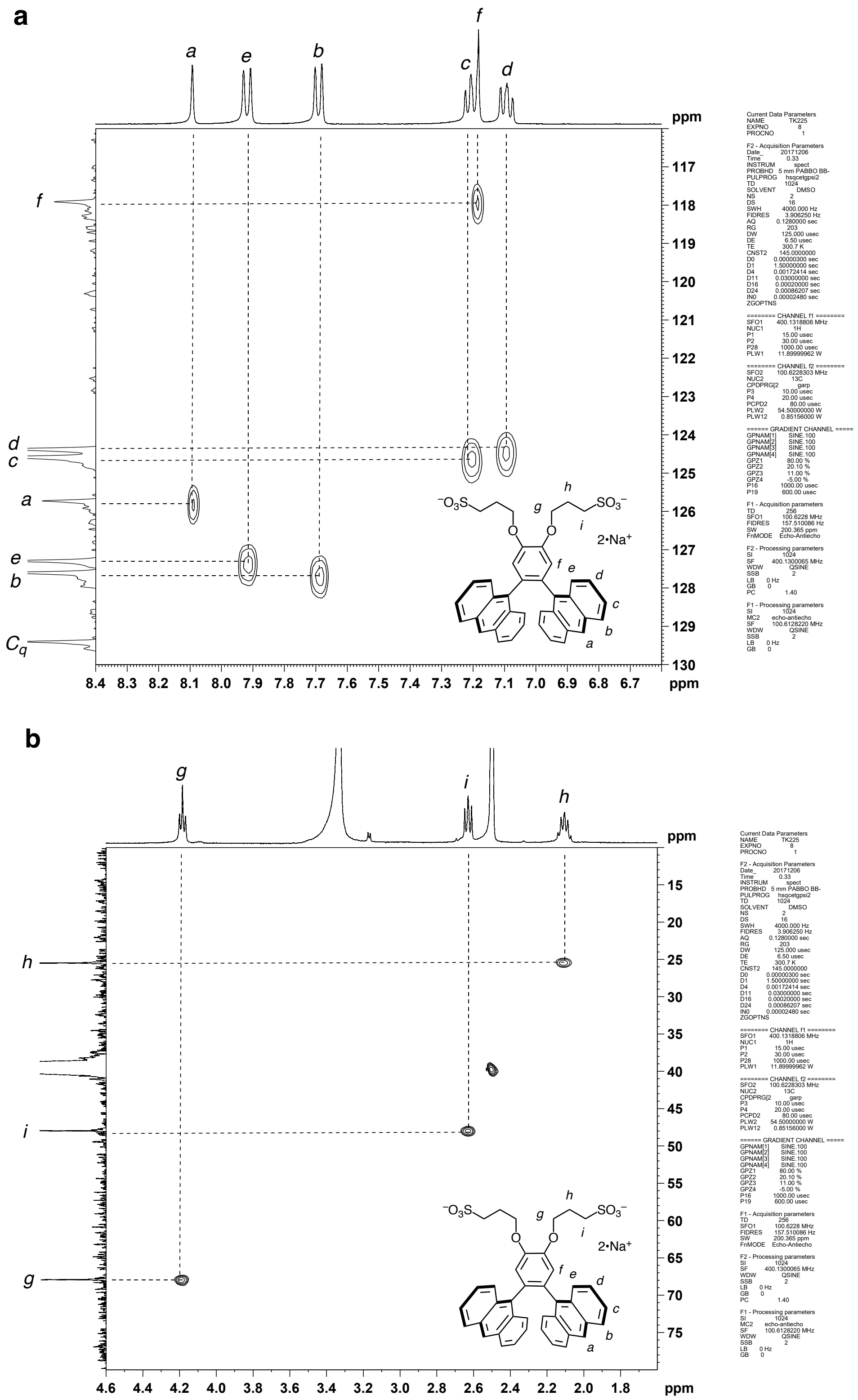

Supplementary Fig. 8 HSQC spectrum (400 MHz, DMSO-d_6, room temperature) of **o-1a** ((a) aromatic and (b) aliphatic regions).

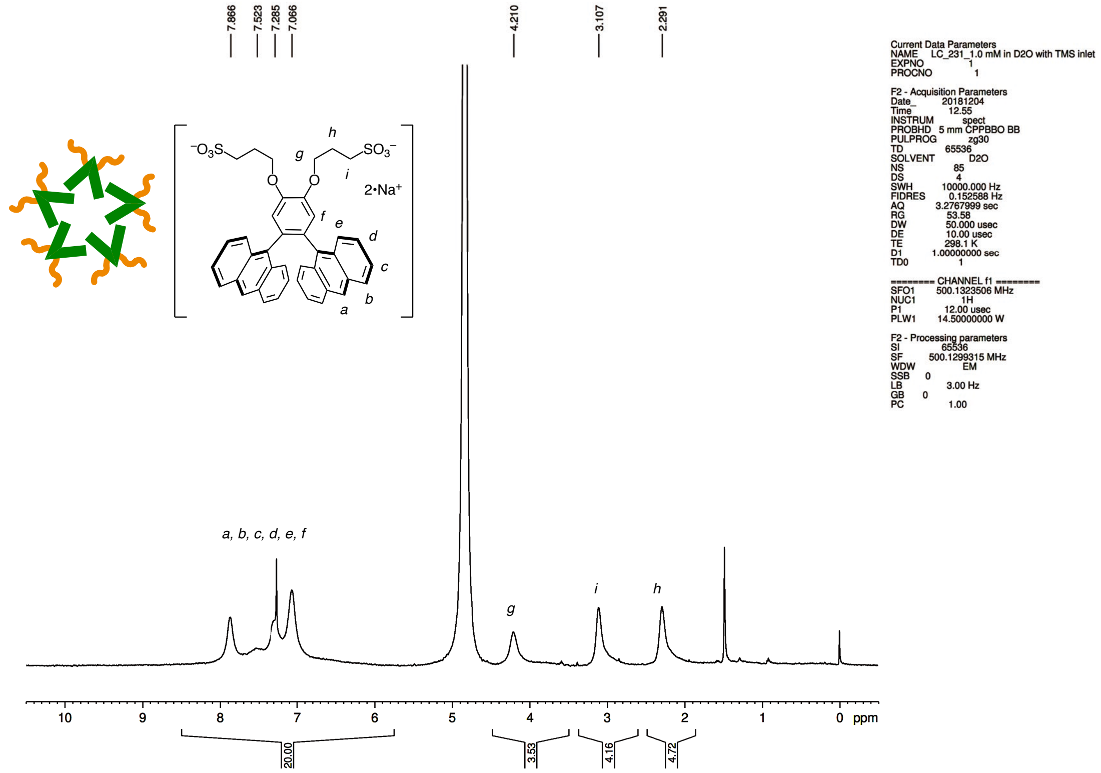

Supplementary Fig. 9 ^{1}H NMR spectrum (500 MHz, D_2O, room temperature) of **2a**.

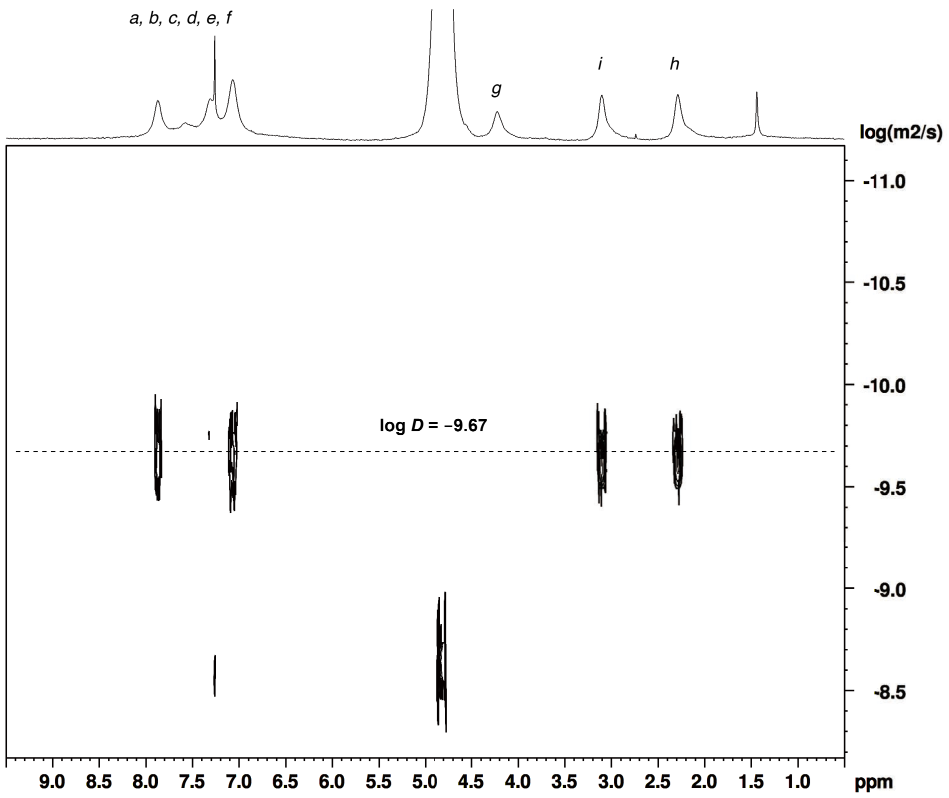

Supplementary Fig. 10 DOSY NMR spectrum (500 MHz, D_2O, 25 ºC) of **2a**.

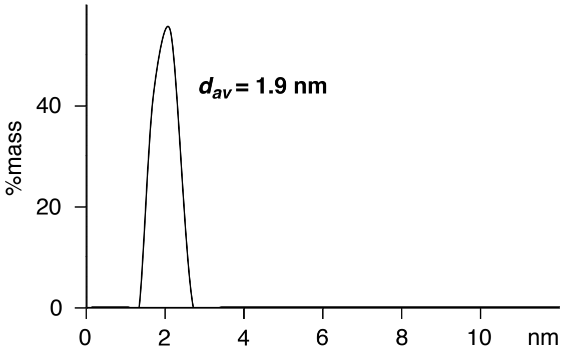

Supplementary Fig. 11 DLS chart (H_2O, 1.0 mM based on **o-1a**, room temperature) of **2a**.

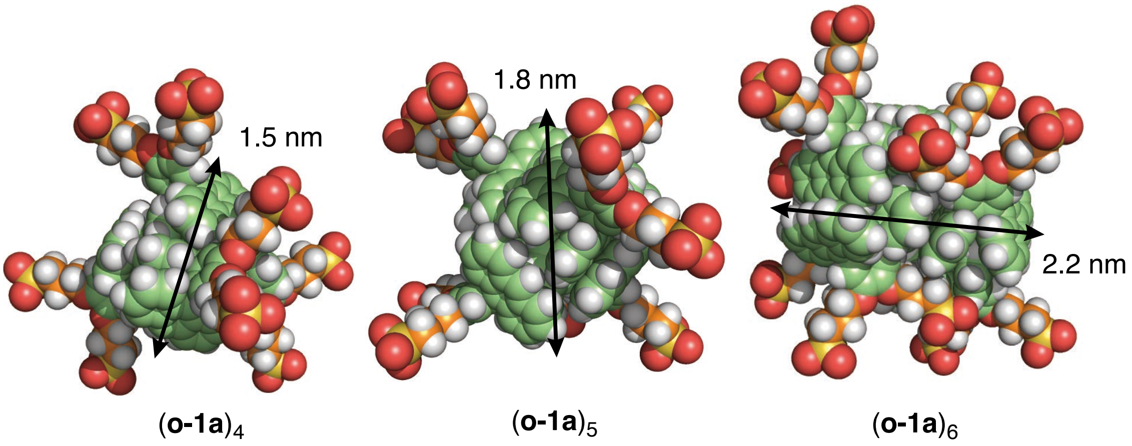

Supplementary Fig. 12 Optimized structures of **2a** composed of (**o-1a**)$_4$, (**o-1a**)$_5$, and (**o-1a**)$_6$ and their average core diameters.

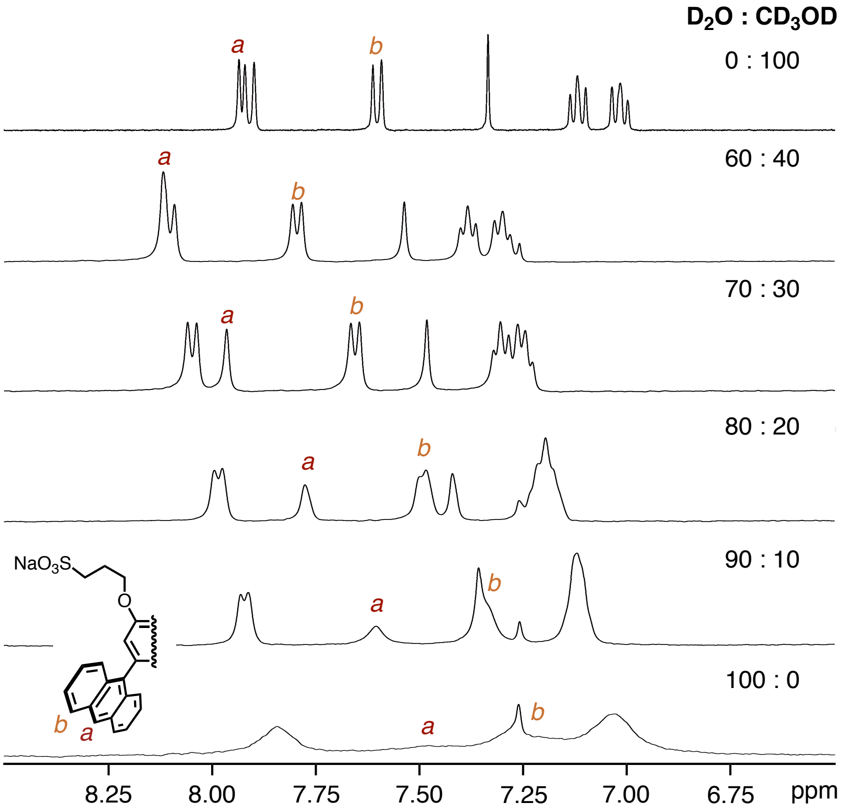

Supplementary Fig. 13 Solvent-dependent ^{1}H NMR spectra (400 MHz, room temperature, 1.0 mM based on **o-1a**) of **2a** in mixed D_2O/CD_3OD solvents. TMS in $CDCl_3$ inlet was used as a calibration standard.

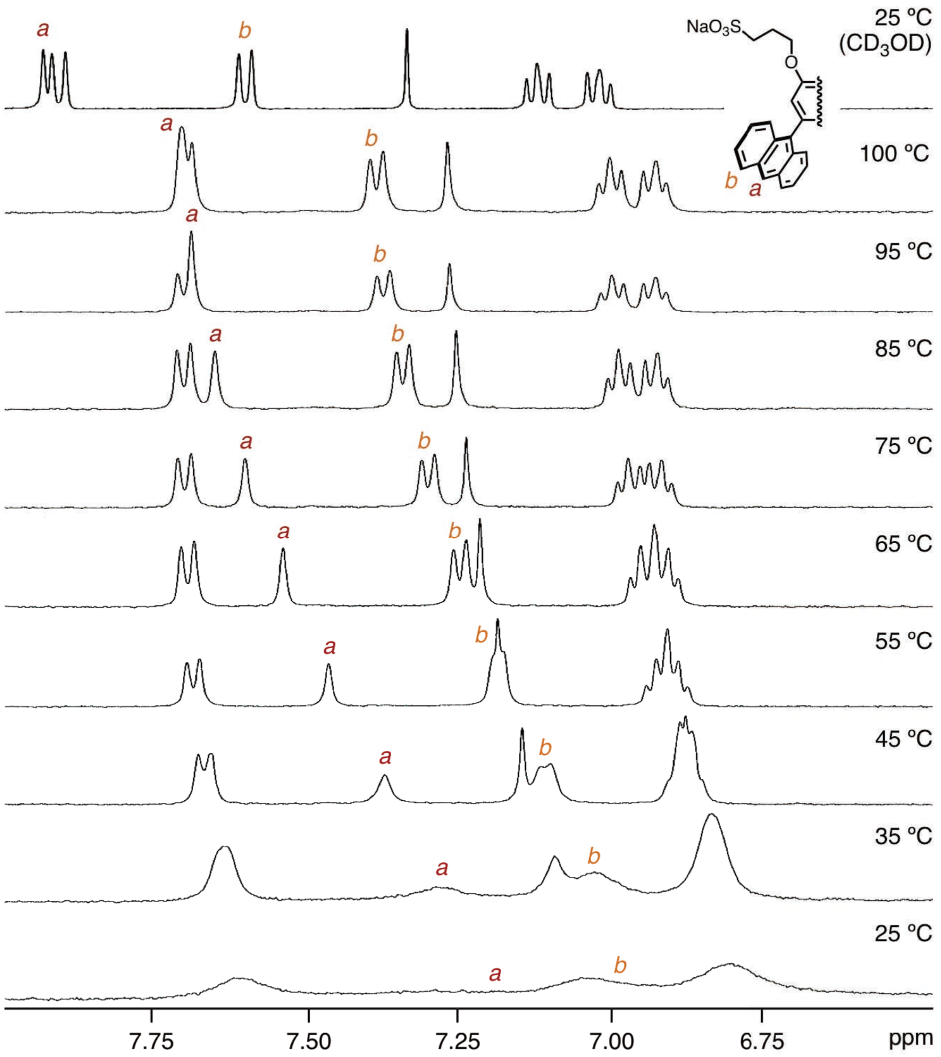

Supplementary Fig. 14 Temperature-dependent ^{1}H NMR spectra (400 MHz, D_2O, 1.0 mM based on **o-1a**) of **2a**. DMSO (1.0 μl) was used as an internal calibration standard.

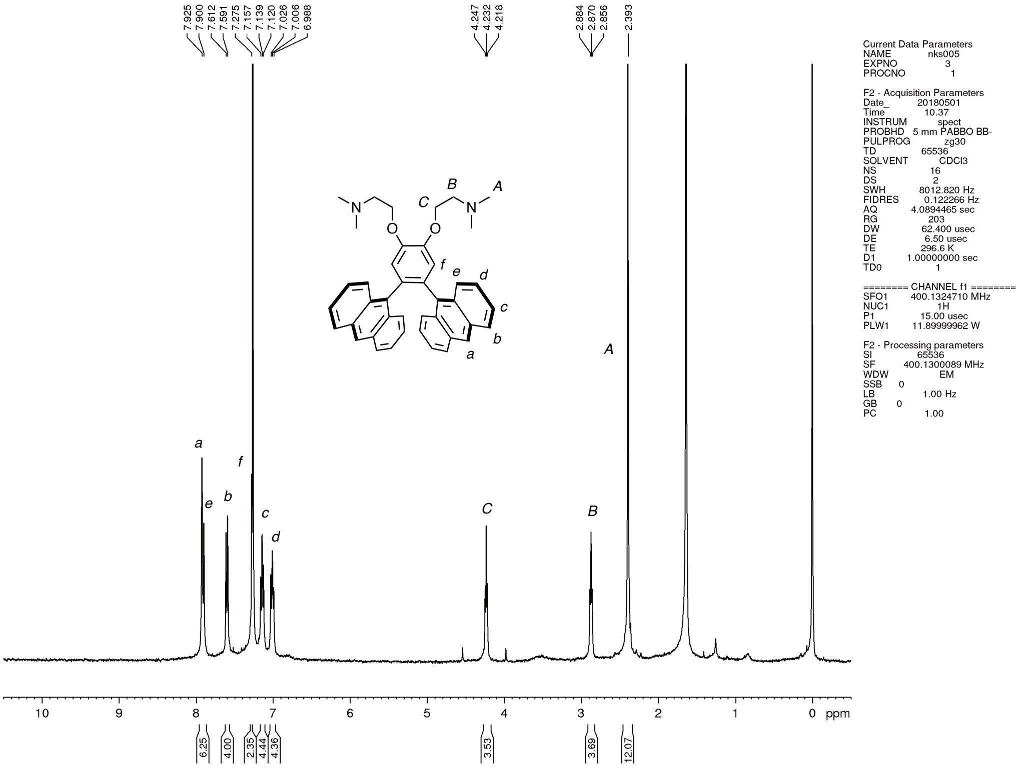

Supplementary Fig. 15 ^{1}H NMR spectrum (400 MHz, $CDCl_3$, room temperature) of $\mathbf{1_{NMe2}}$.

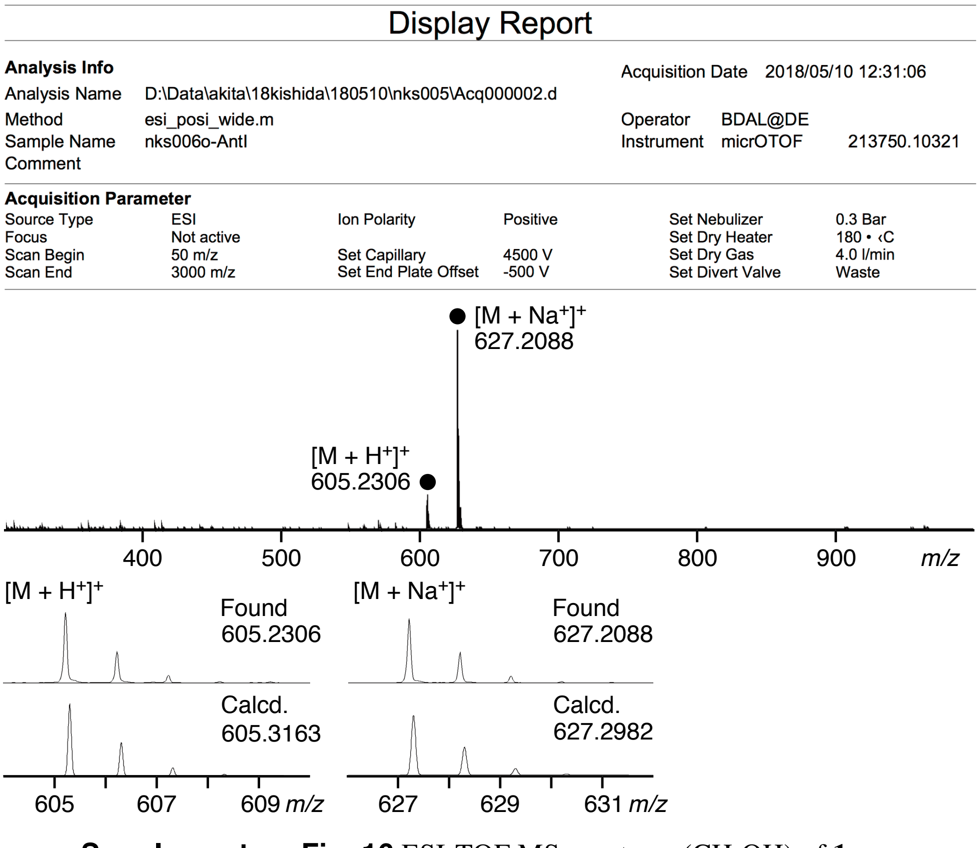

Supplementary Fig. 16 ESI-TOF MS spectrum (CH_3OH) of $\mathbf{1_{NMe2}}$.

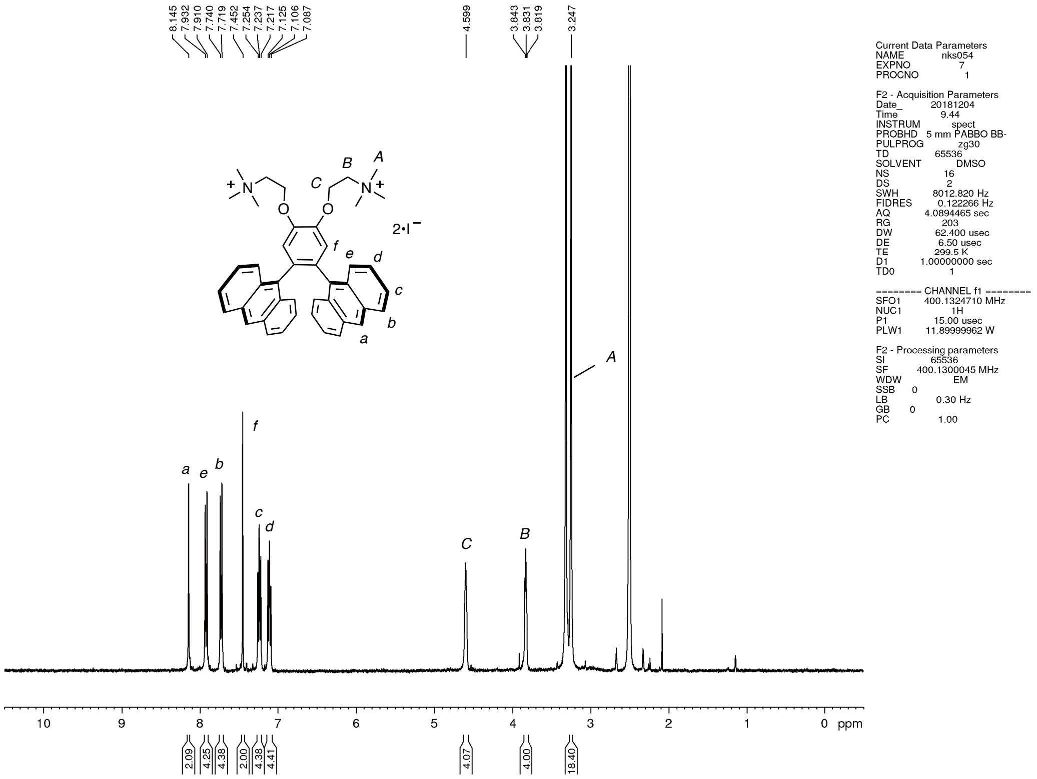

Supplementary Fig. 17 ^{1}H NMR spectrum (400 MHz, DMSO-d_6, room temperature) of $\mathbf{1_{NMe3}}$.

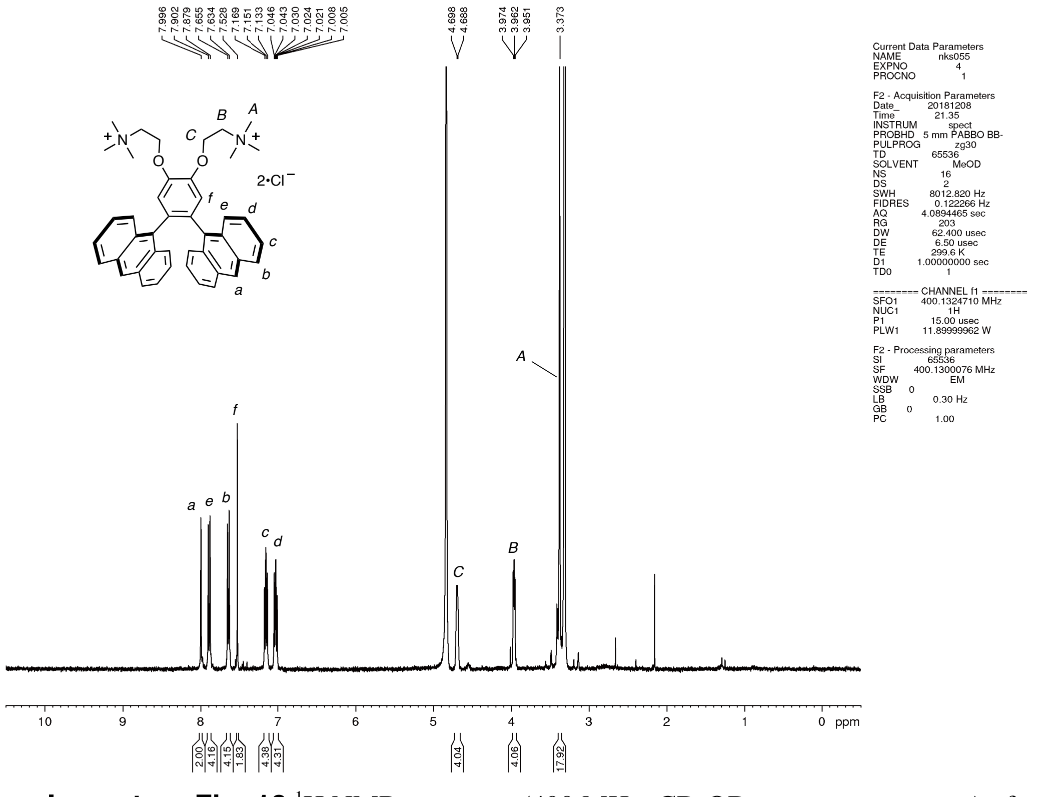

Supplementary Fig. 18 ^{1}H NMR spectrum (400 MHz, CD_3OD, room temperature) of **o-1b**.

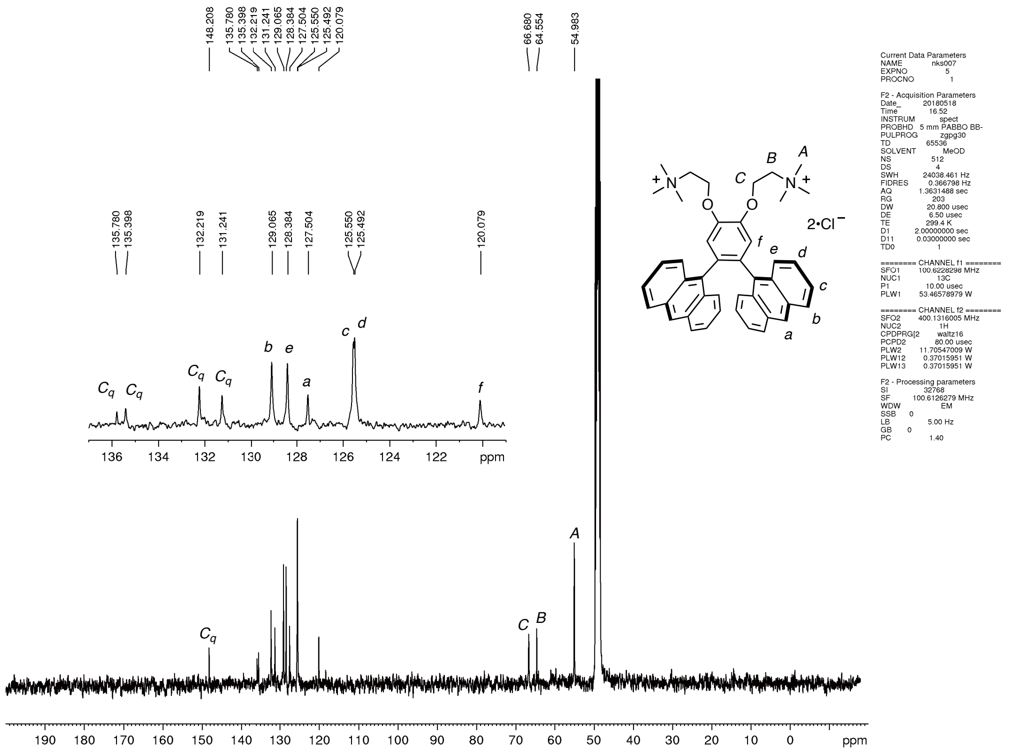

Supplementary Fig. 19 ^{13}C NMR spectrum (100 MHz, CD_3OD, room temperature) of **o-1b**.

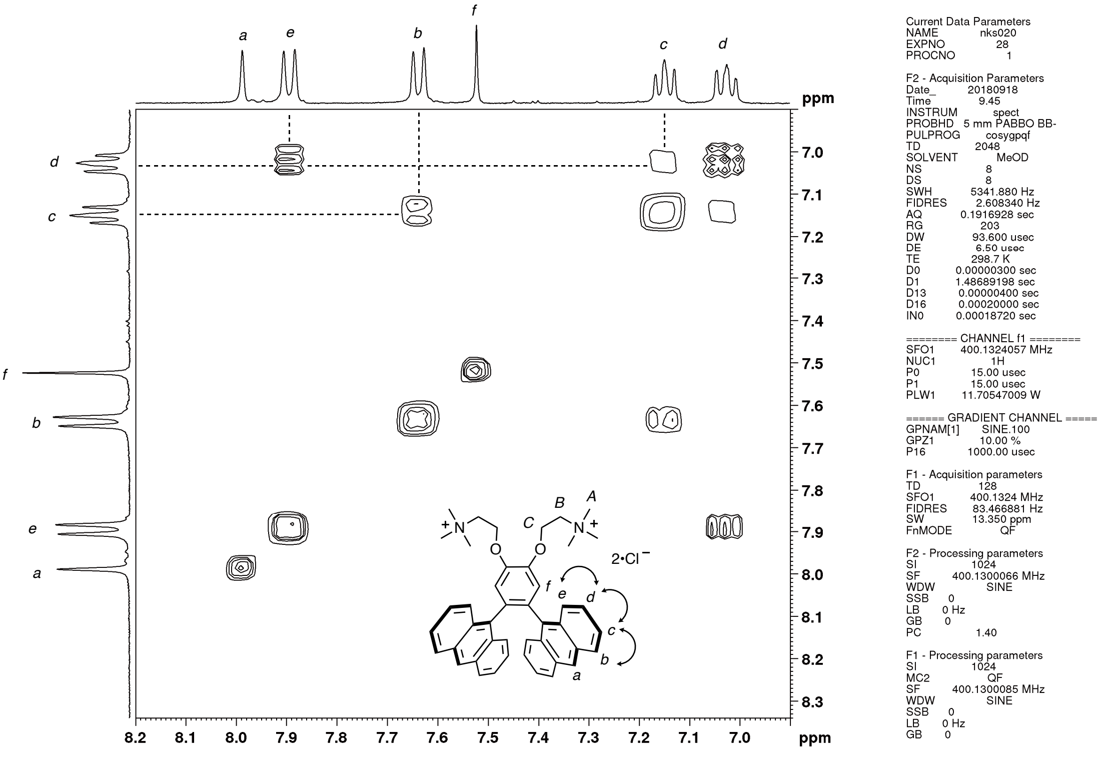

Supplementary Fig. 20 ^{1}H-^{1}H COSY spectrum (400 MHz, CD_3OD, room temperature) of **o-1b**.

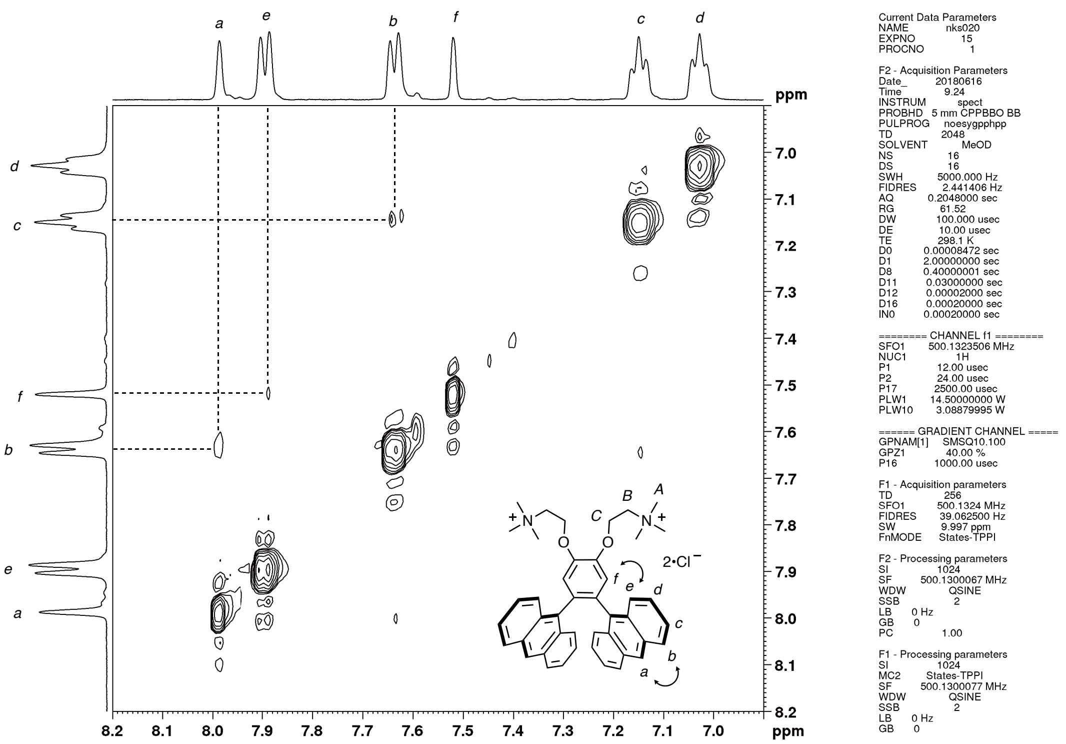

Supplementary Fig. 21 NOESY spectrum (500 MHz, CD_3OD, room temperature) of **o-1b**.

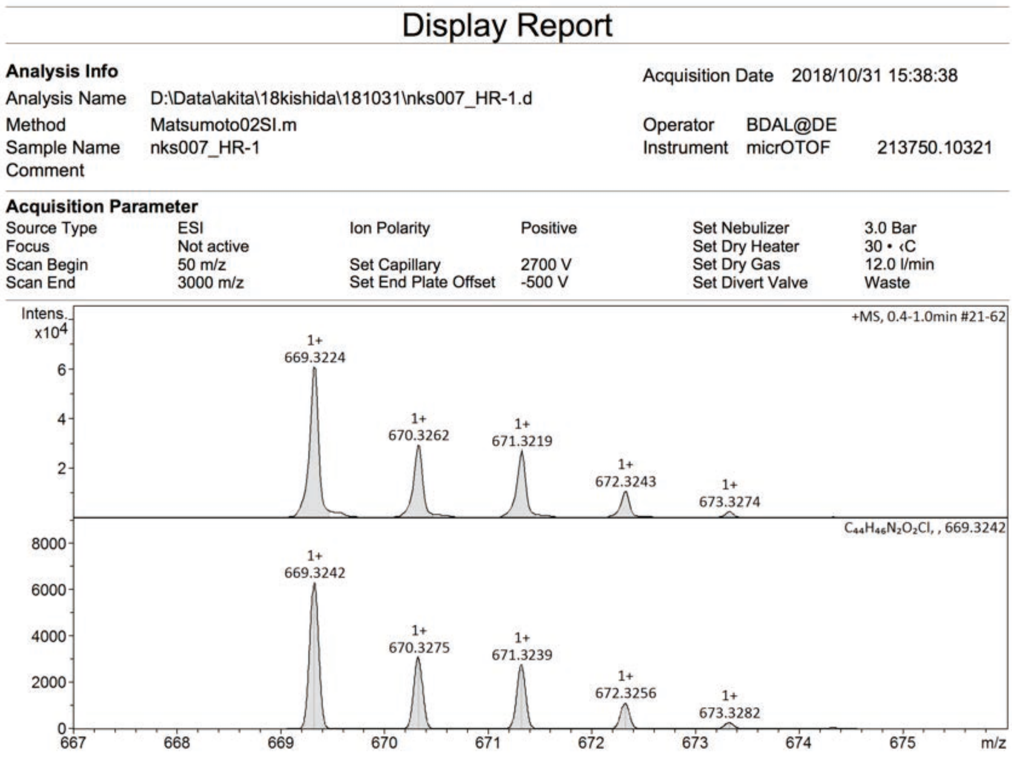

Supplementary Fig. 22 HR MS spectrum (ESI, CH_3OH) of **o-1b**.

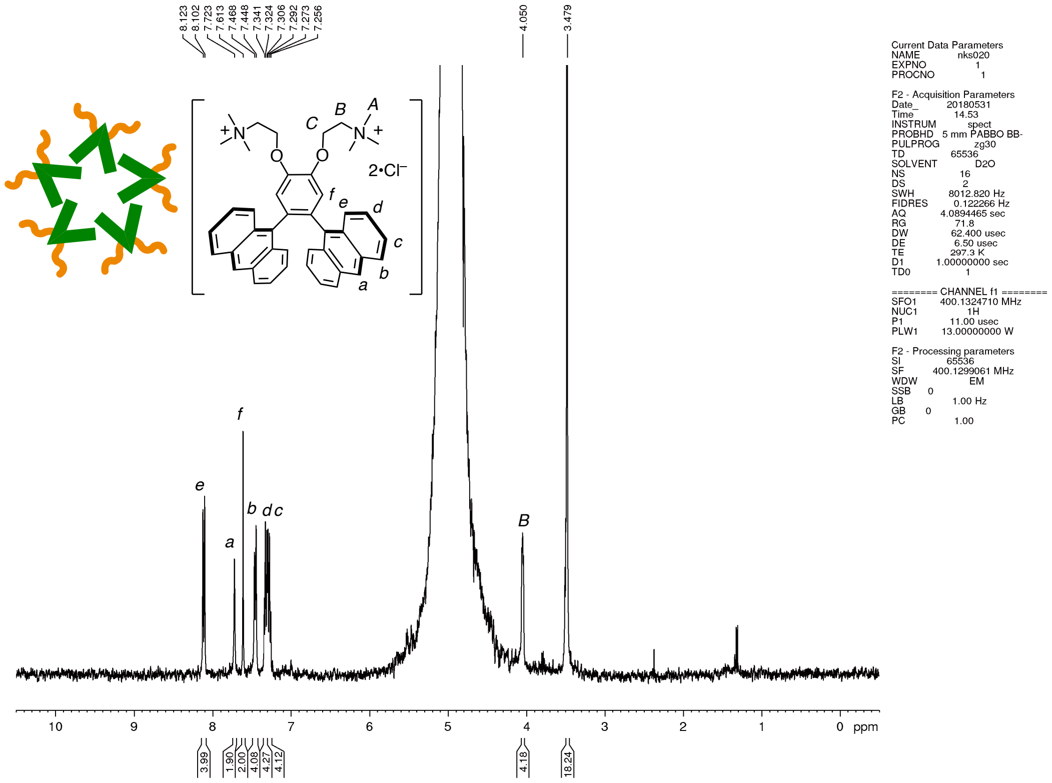

Supplementary Fig. 23 ^{1}H NMR spectrum (400 MHz, D_2O, room temperature) of **2b**.

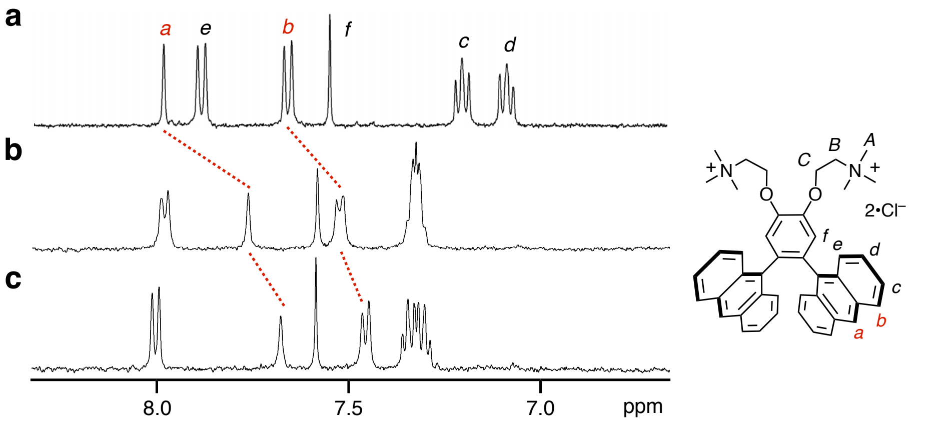

Supplementary Fig. 24 Solvent-dependent ^{1}H NMR spectra (400 MHz, room temperature, TMS as an external standard) of **o-1b** in CD_3OD/D_2O (v/v) = (a) 100:0, (b) 3:97, and (c) 0:100.

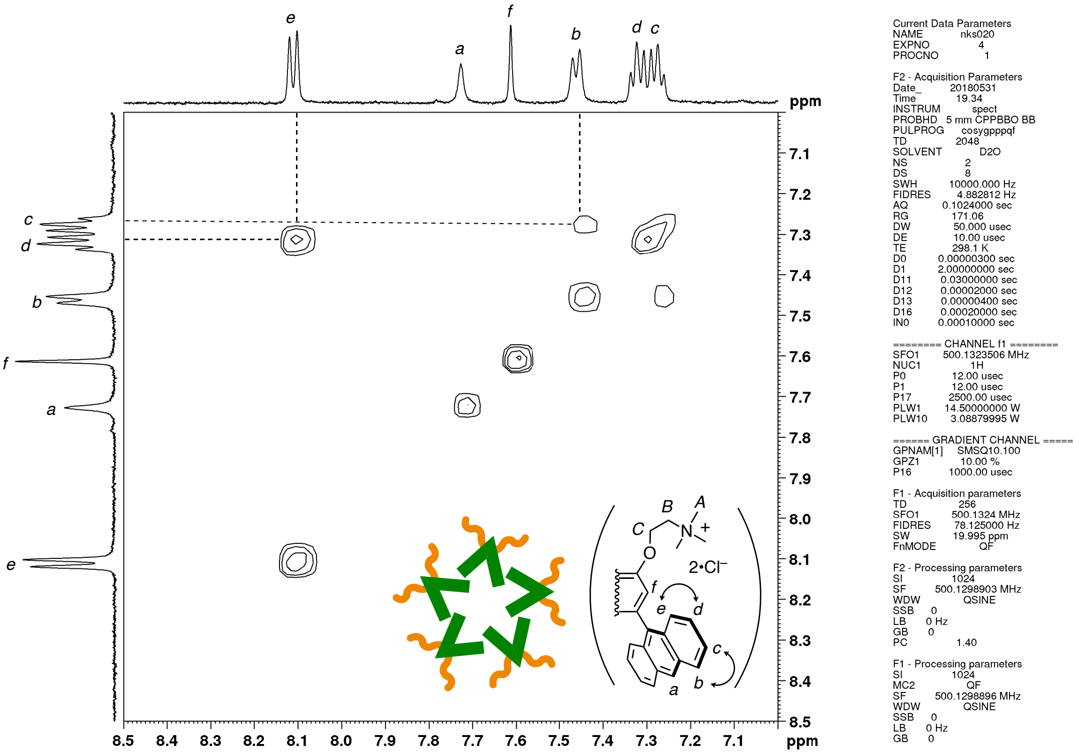

Supplementary Fig. 25 ^{1}H-^{1}H COSY spectrum (500 MHz, D_2O, room temperature) of **2b**.

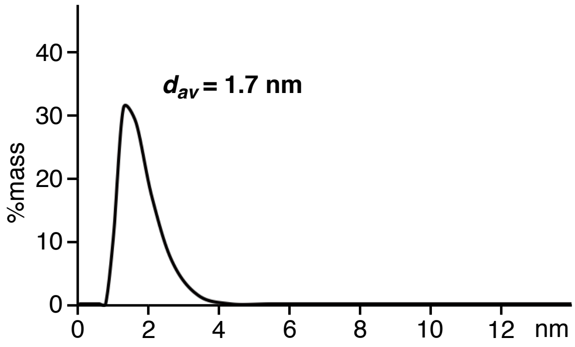

Supplementary Fig. 26 DLS chart (H_2O, 0.1 mM based on **o-1b**, room temperature) of **2b**.

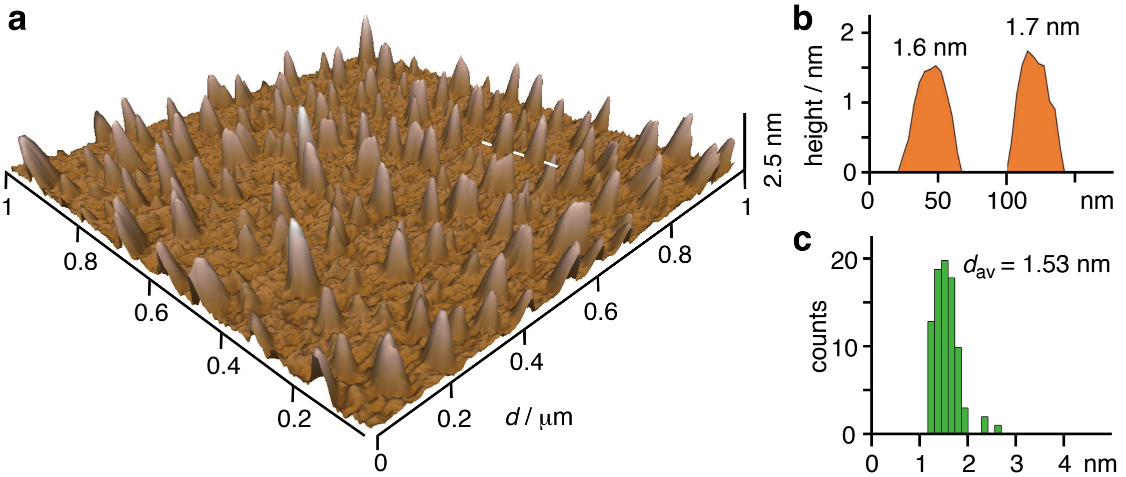

Supplementary Fig. 27 (a) AFM image (dry, mica, room temperature) of **2b**, and (b) the height profile of the selected feature and (c) the size and number distribution.

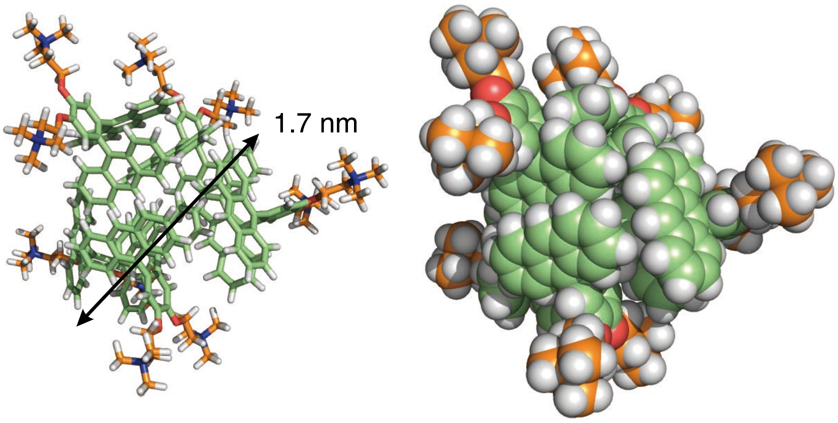

Supplementary Fig. 28 Optimized structure of **2b** composed of (**o-1b**)$_5$.

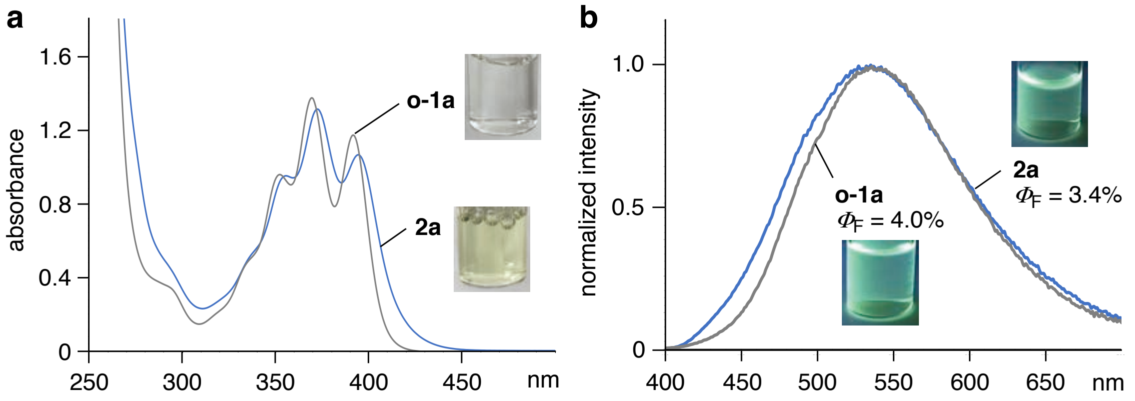

Supplementary Fig. 29 (a) UV-vis and (b) fluorescence spectra (1.0 mM based on **o-1a**, room temperature, λ_{ex} = 370 nm) of **o-1a** in CH_3OH and **2a** in H_2O, and their photographs (λ_{ex} = 365 nm for **o-1a** and **2a**).

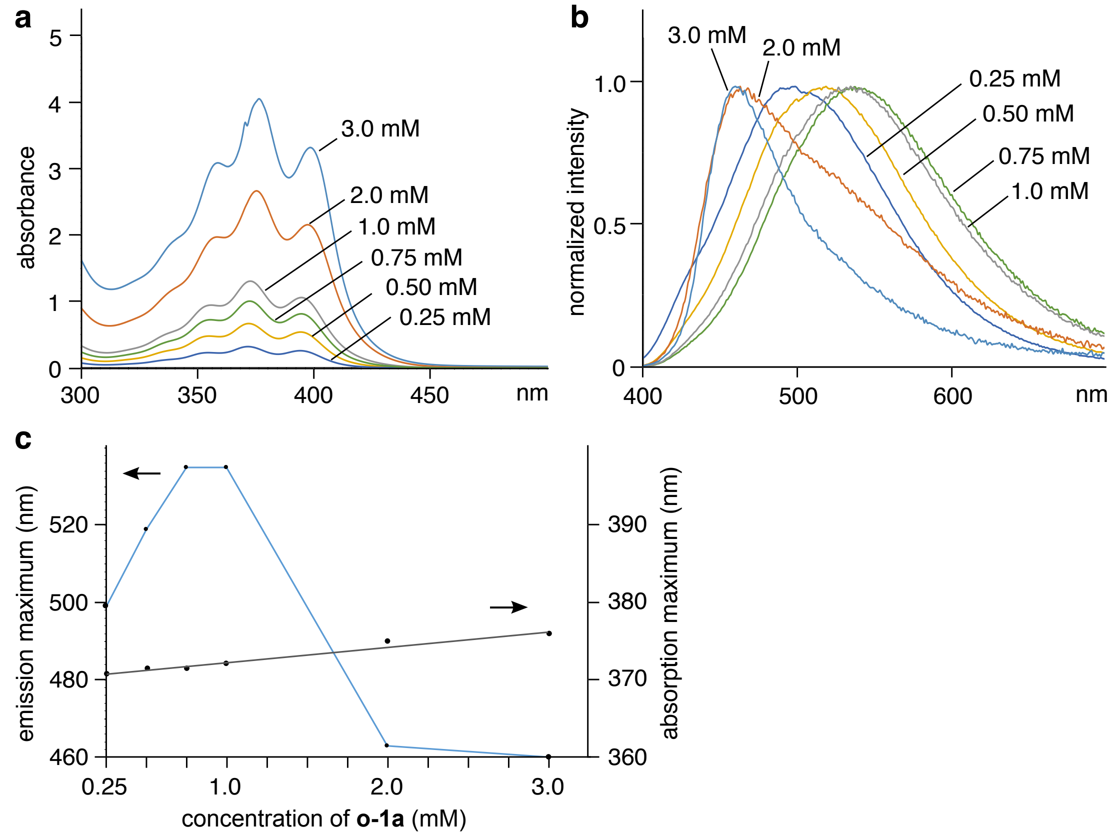

Supplementary Fig. 30 Concentration-dependent (a) UV-vis and (b) fluorescence spectra (H_2O, room temperature, λ_{ex} = 370 nm) of **o-1a**. (c) Concentration-dependent shift of the emission and absorption maximums of **o-1a** in H_2O (λ_{ex} = 370 nm).

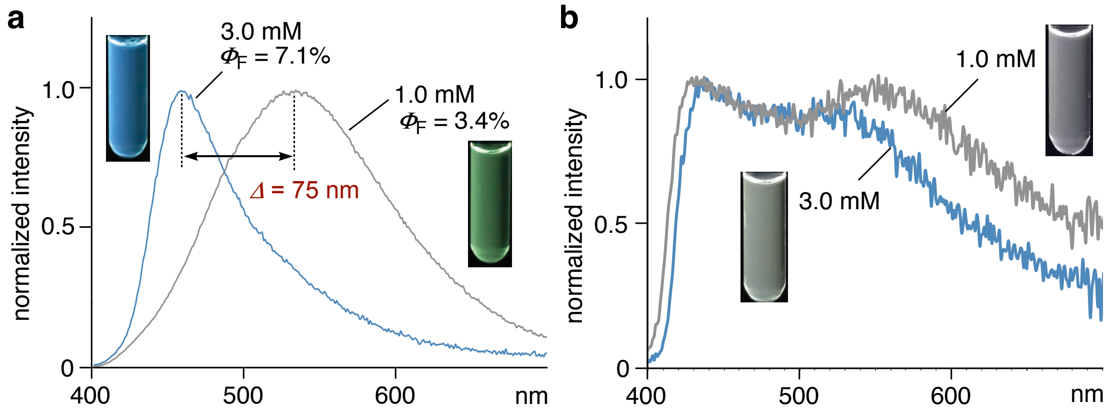

Supplementary Fig. 31 Fluorescence spectra (H_2O, room temperature, λ_{ex} = 370 nm) of freshly filtered 1.0 and 3.0 mM solutions of (a) **o-1a** and (b) **3**, and their photographs (λ_{ex} = 365 nm).

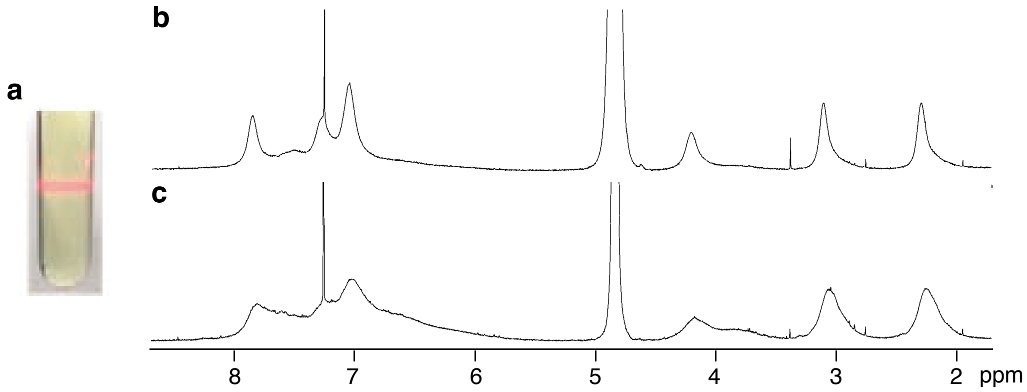

Supplementary Fig. 32 (a) Tyndall effect of the 3.0 mM solution of **o-1a** in H_2O under irradiation with a red laser pointer. ^1H NMR spectra (500 MHz, room temperature, TMS as an external standard) of a (b) 1.0 mM and (c) 3.0 mM solution of **o-1a** in D_2O.

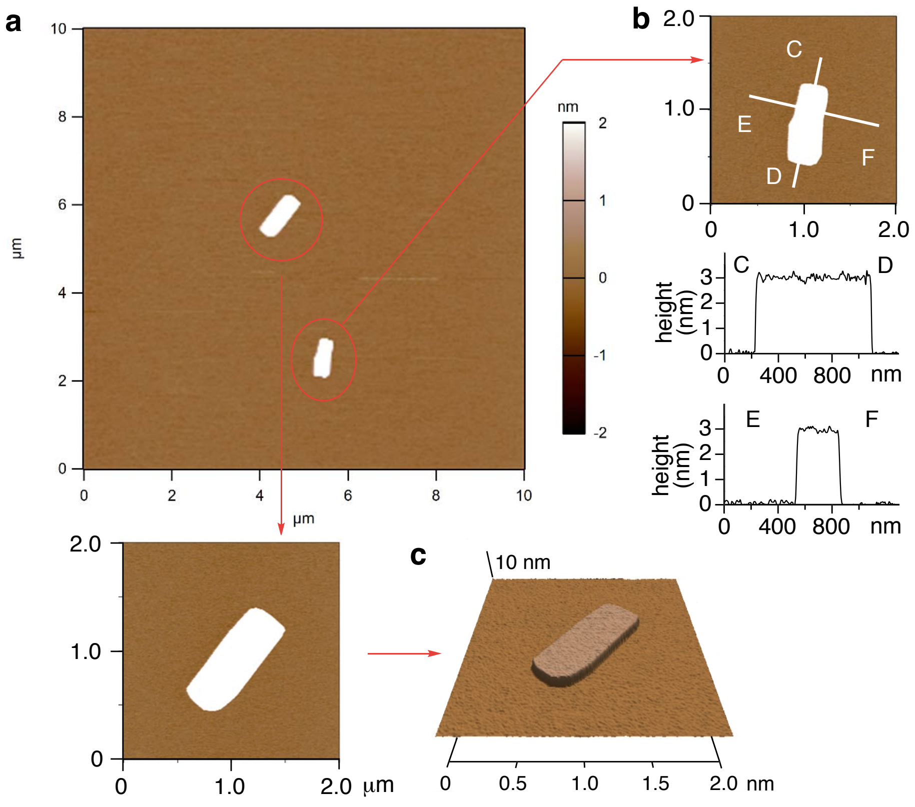

Supplementary Fig. 33 (a) Representative AFM image (room temperature, wet, mica) of a 3.0 mM solution of **o-1a** in H_2O, (b) including the height profiles of one of the rectangular sheets, and (c) a 3D representation.

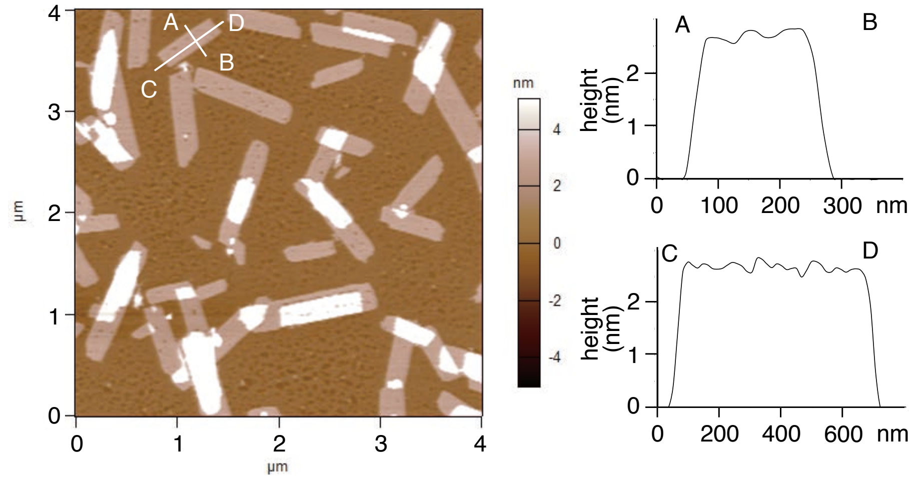

Supplementary Fig. 34 Representative AFM image (room temperature, dry, mica) of a 3.0 mM solution of **o-1a** in H_2O, including the height profiles of one of the rectangular sheets.

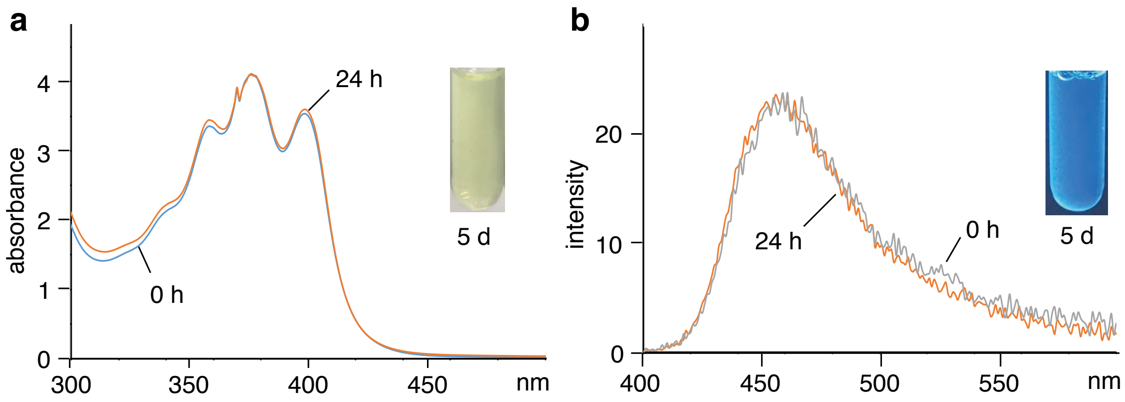

Supplementary Fig. 35 (a) UV-vis and (b) fluorescence spectra (3.0 mM based on **o-1a**, room temperature, λ_{ex} = 370 nm) directly and 24 h after filtration using a membrane filter (pore size: 200 nm), and photographs of the solution after 5 d (right: λ_{ex} = 365 nm).

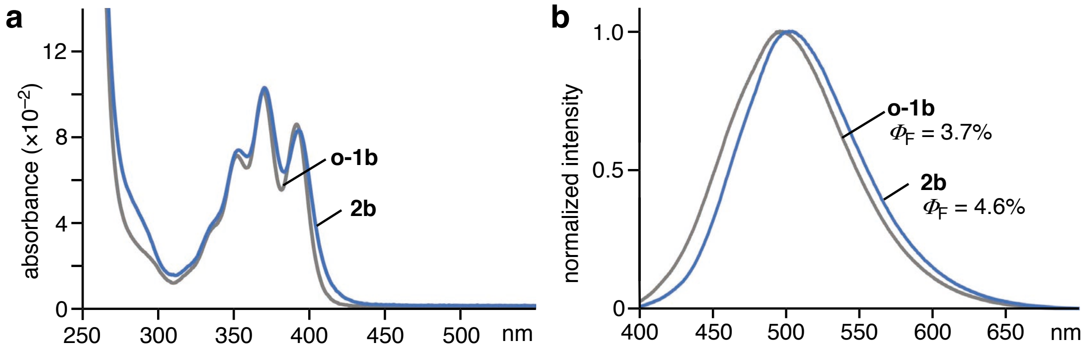

Supplementary Fig. 36 (a) UV-vis and (b) fluorescence spectra (0.1 mM based on **o-1b**, room temperature, λ_{ex} = 370 nm) of **o-1b** in CH_3OH and **2b** in H_2O.

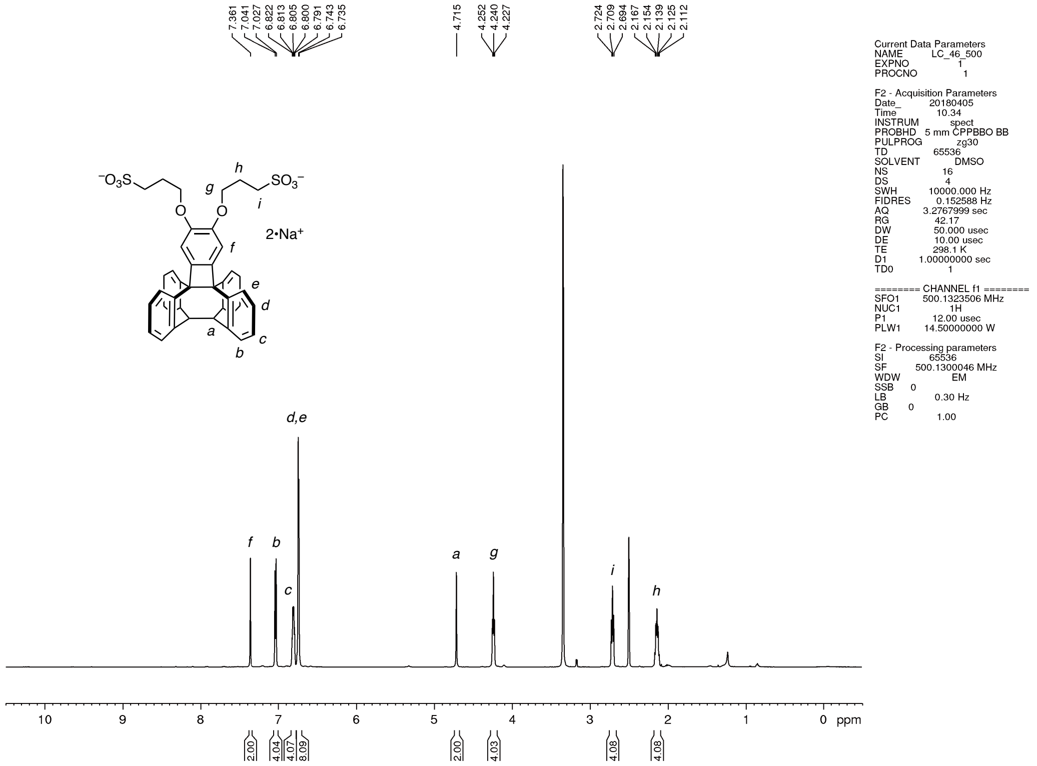

Supplementary Fig. 37 ^{1}H NMR spectrum (500 MHz, DMSO-d_6, room temperature) of **c-1a**.

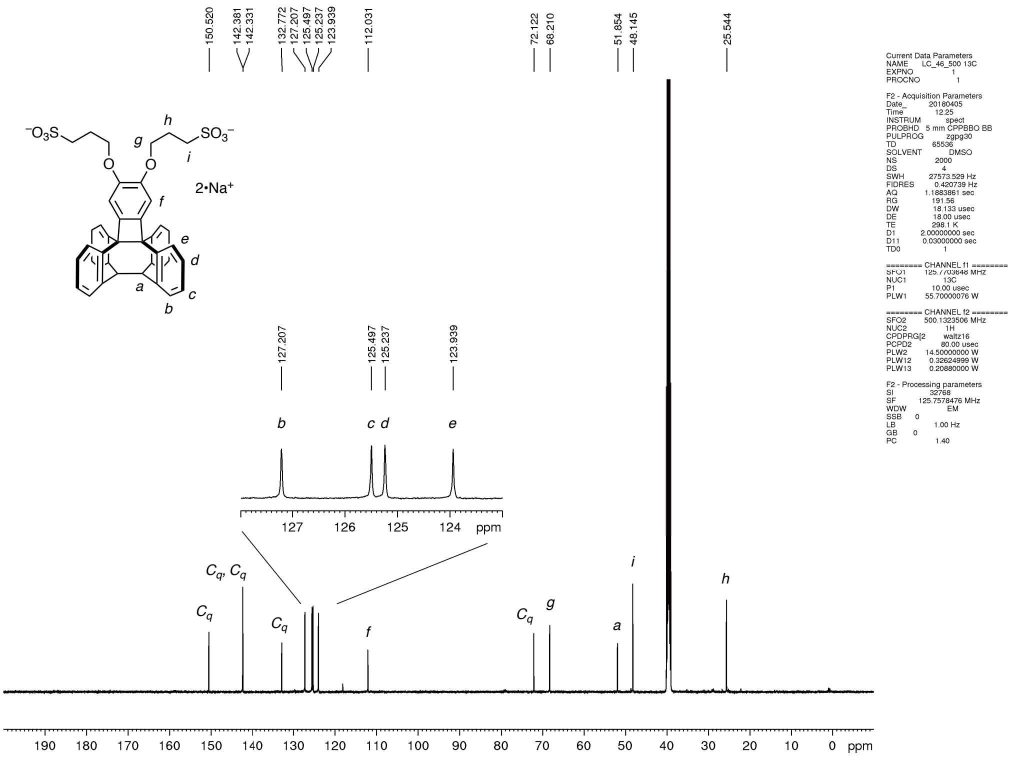

Supplementary Fig. 38 ^{13}C NMR spectrum (125 MHz, DMSO-d_6, room temperature) of **c-1a**.

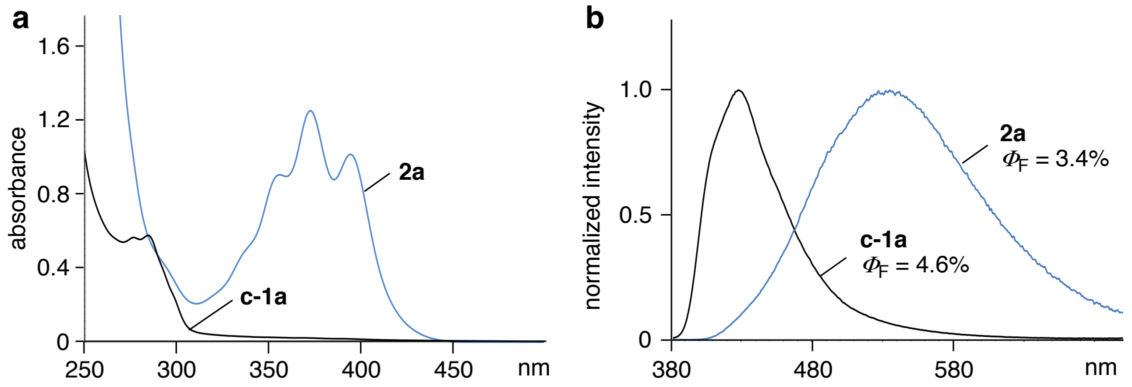

Supplementary Fig. 39 (a) UV-vis and (b) fluorescence spectra (H_2O, 1.0 mM based on **o-1a**, room temperature, λ_{ex} = 370 nm) of **2a** and **c-1a**, generated by light irradiation at 380 nm for 5 min.

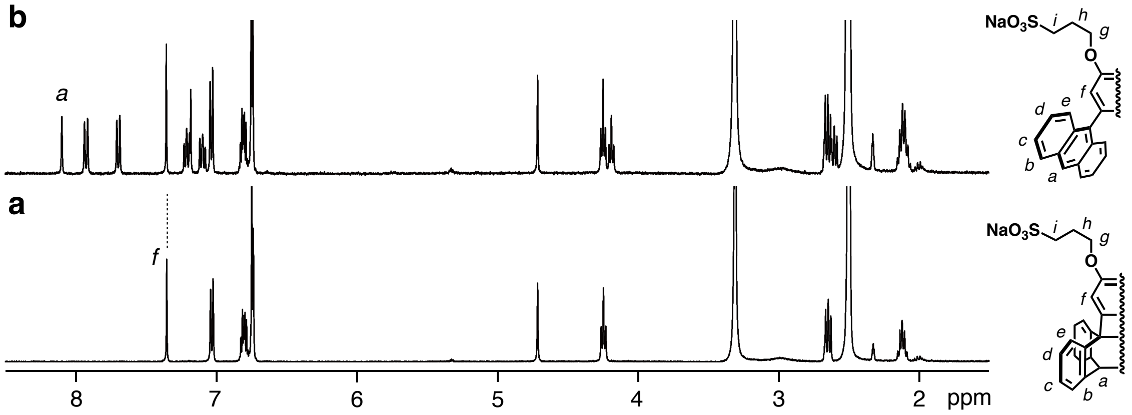

Supplementary Fig. 40 ^{1}H NMR spectra (400 MHz, DMSO-d_6, room temperature, 1.0 mM) of **c-1a** (a) before and (b) after light irradiation at 287 nm for 25 min.

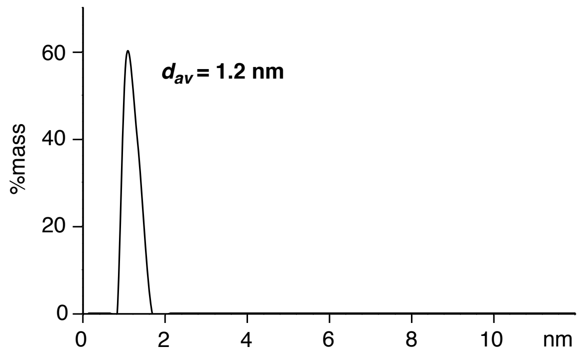

Supplementary Fig. 41 DLS chart of **c-1a** (H_2O, 1.0 mM, room temperature).

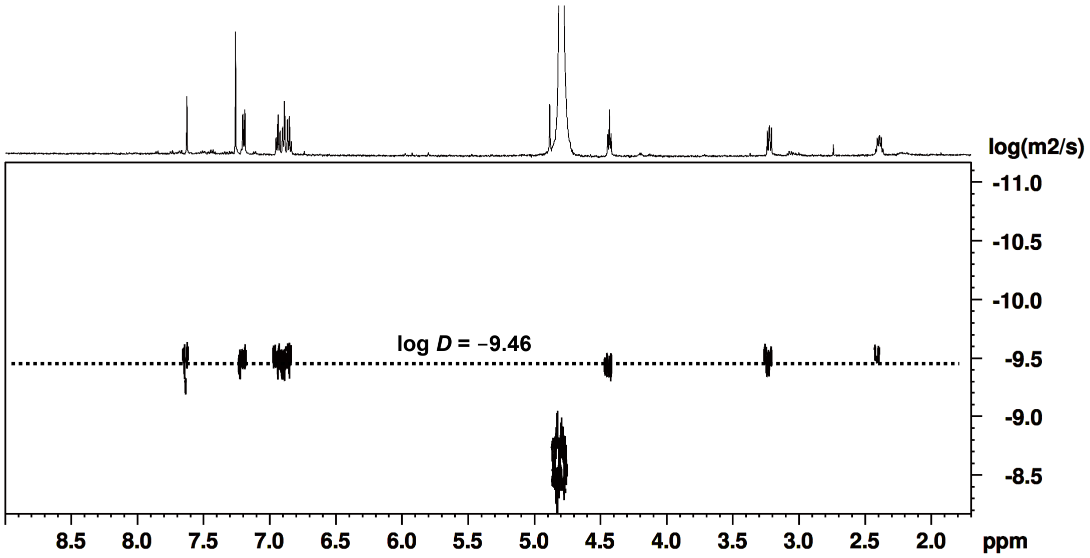

Supplementary Fig. 42 DOSY NMR spectrum (500 MHz, 1.0 mM, D_2O, 25 °C) of **c-1a**.

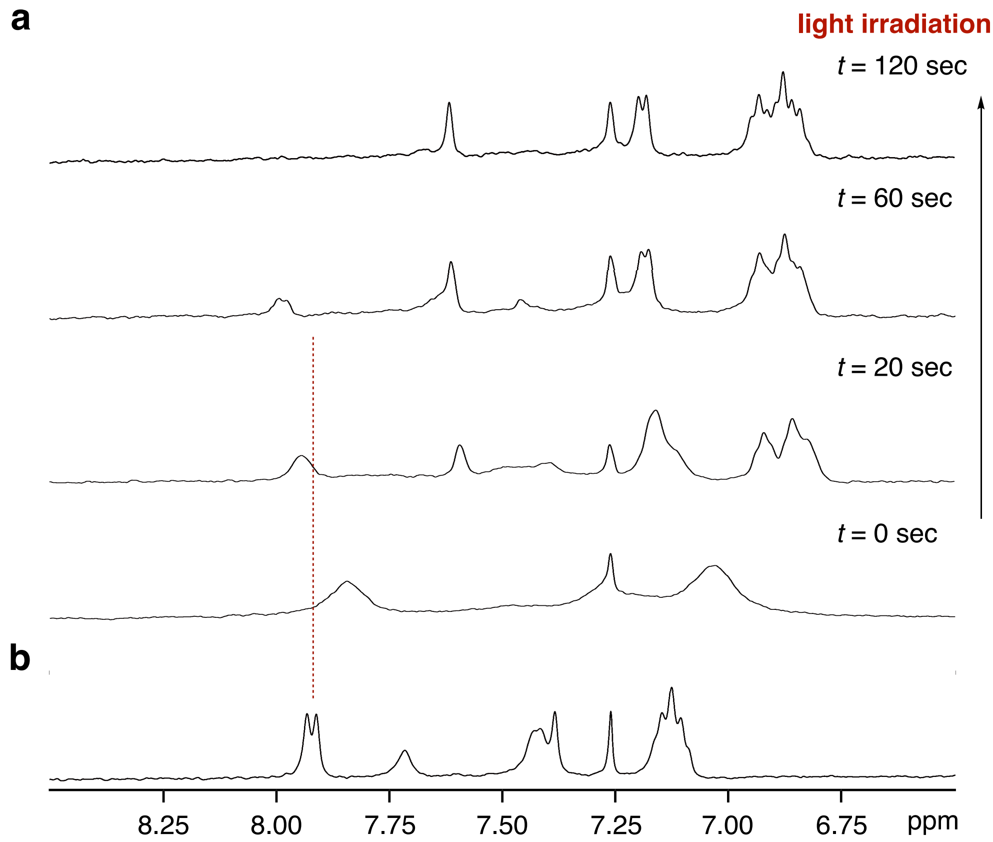

Supplementary Fig. 43 (a) ^{1}H NMR spectra (400 MHz, D_2O, room temperature, 1.0 mM based on **o-1a**) of **2a** after a stepwise irradiation with 380 nm UV light (3 W × 2) at room temperature. A capillary with TMS in $CDCl_3$ was used as a calibration standard. (b) ^{1}H NMR spectrum (400 MHz, D_2O, room temperature, 0.5 mM based on **o-1a**) of **2a**.

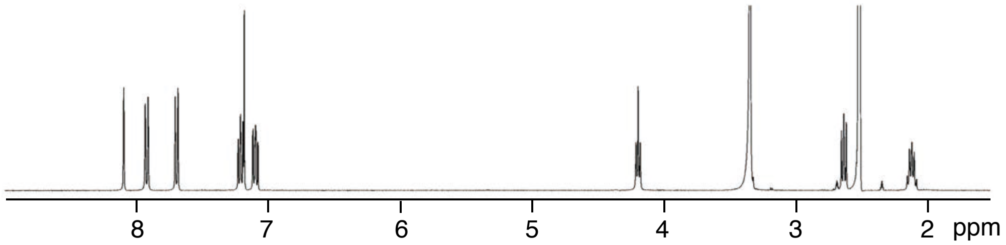

Supplementary Fig. 44 ^{1}H NMR spectrum (400 MHz, DMSO-d_6, room temperature) of **o-1a** after 10 min irradiation at 380 nm in the solid state.

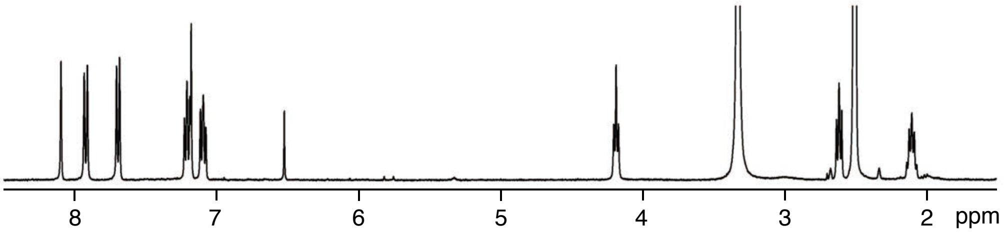

Supplementary Fig. 45 ^{1}H NMR spectrum (400 MHz, DMSO-d_6, room temperature) of **o-1a**, obtained after lyophilization of the aqueous solution after the fifth reopening via microwave heating.

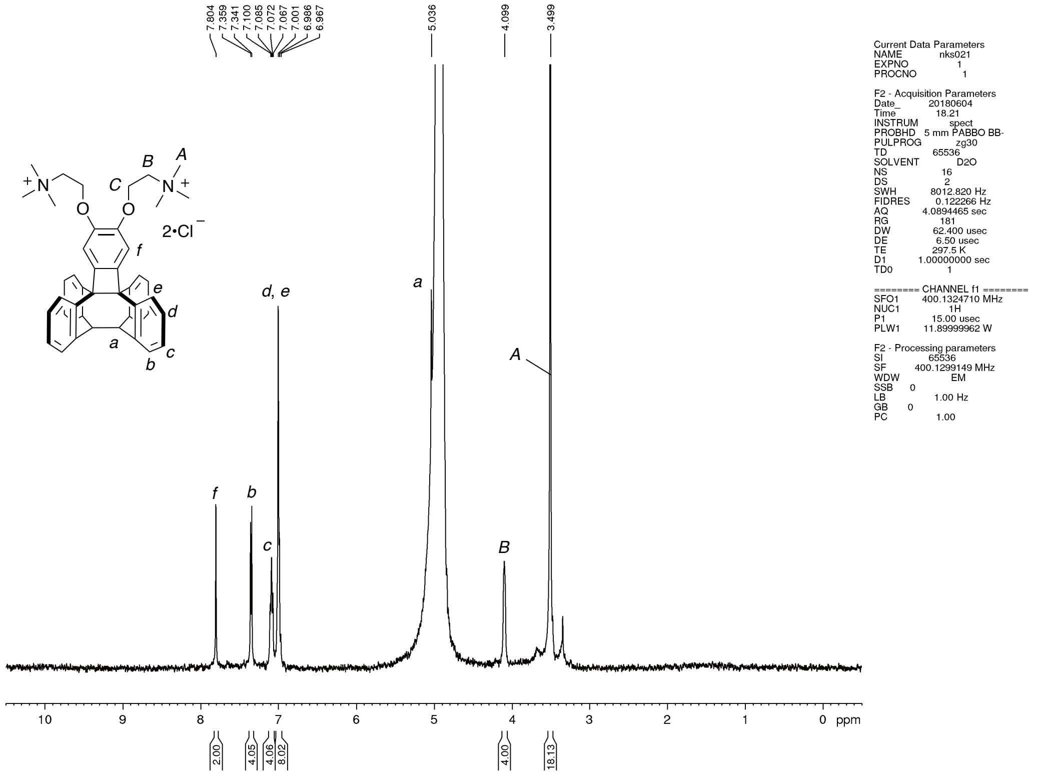

Supplementary Fig. 46 ^{1}H NMR spectrum (400 MHz, D_2O, room temperature) of **c-1b**.

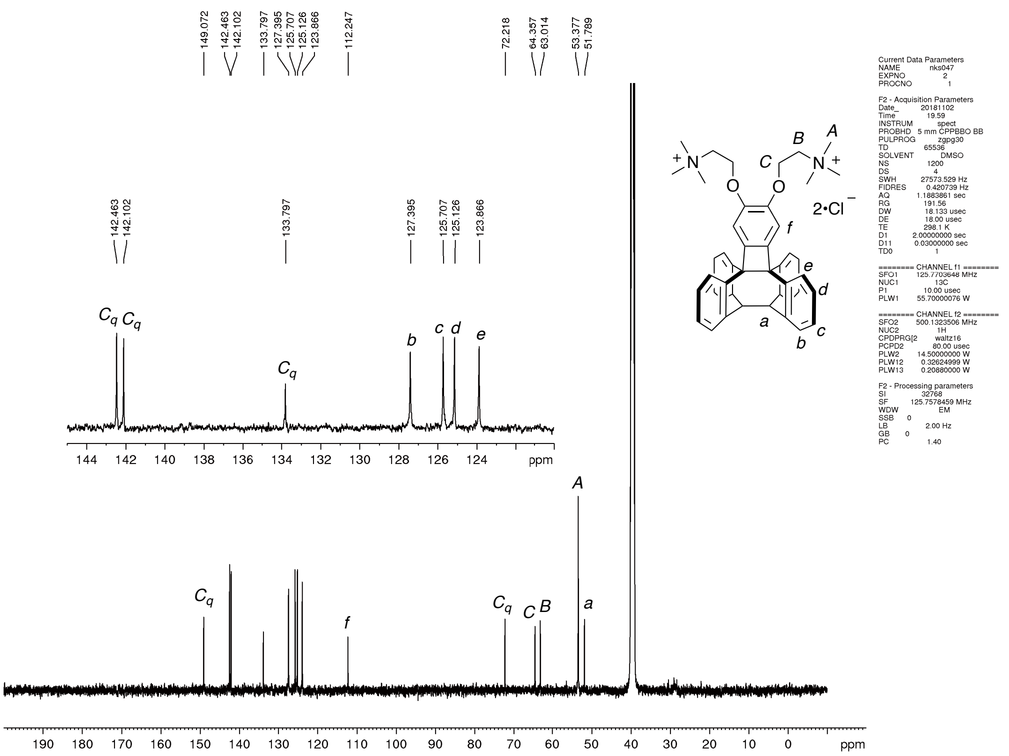

Supplementary Fig. 47 ^{13}C NMR spectrum (125 MHz, DMSO-d_6, room temperature) of **c-1b**.

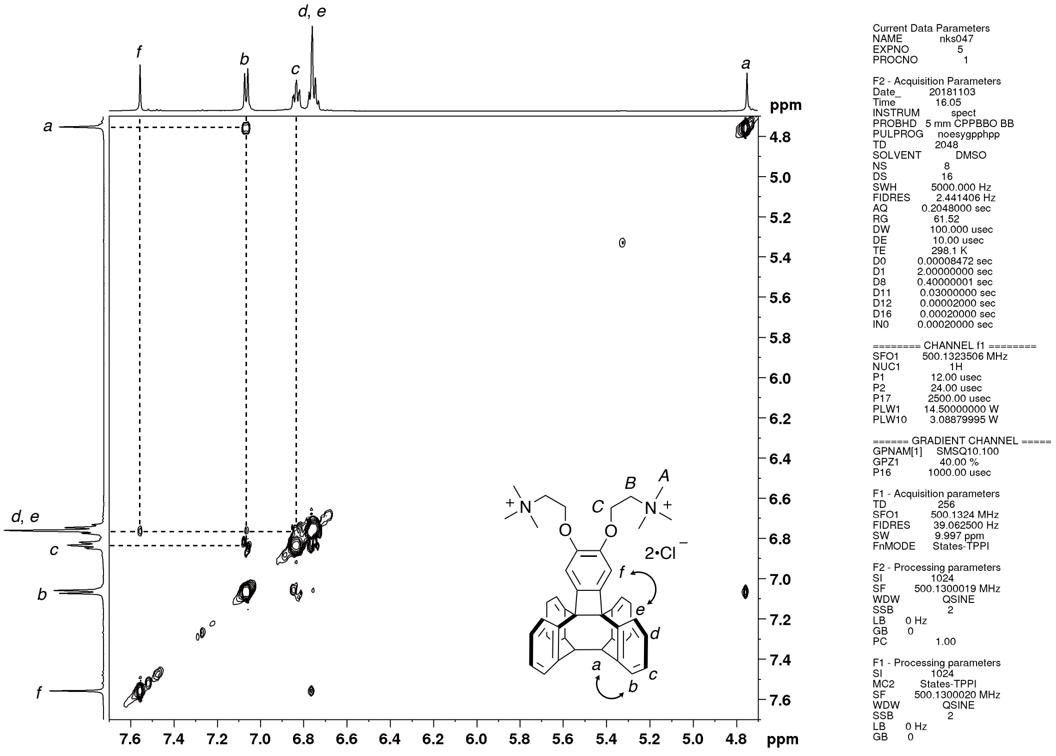

Supplementary Fig. 48 NOESY spectrum (500 MHz, DMSO-d_6, room temperature) of **c-1b**.

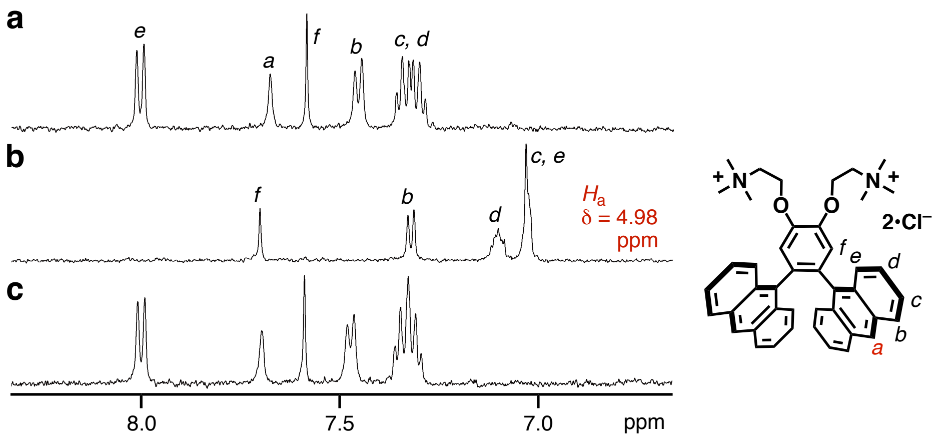

Supplementary Fig. 49 ^{1}H NMR spectra (400 MHz, D_2O, room temperature, TMS as an external standard) of **2b** (a) before and (b) after UV-light irradiation (λ = 380 nm) for 7 min, and then (c) the product after heating at 160 ºC for 30 min.

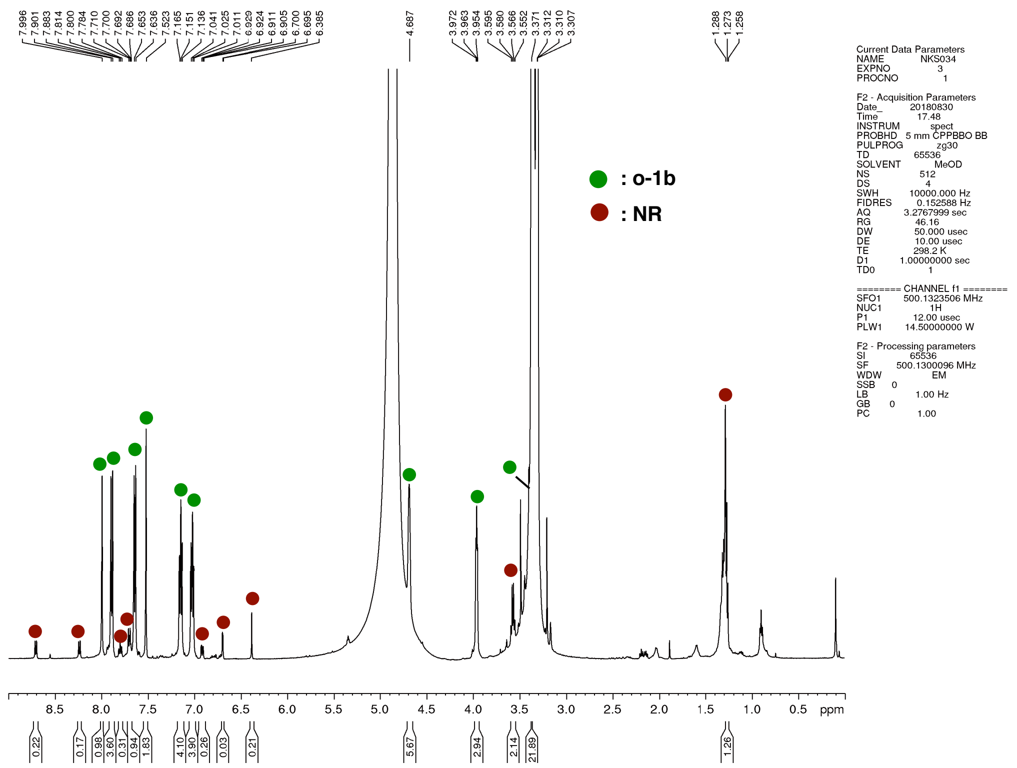

Supplementary Fig. 50 ^{1}H NMR spectrum (500 MHz, CD_3OD, room temperature) of **2b•(NR)**$_2$ obtained after lyophilization of the aqueous solution.

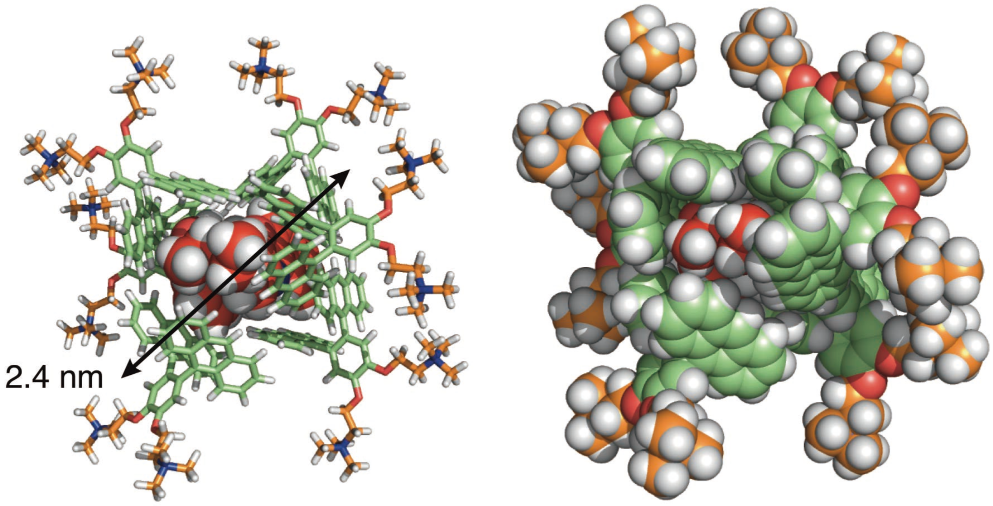

Supplementary Fig. 51 Optimized structure of $(\mathbf{o\text{-}1b})_6$•$(\mathbf{NR})_2$ including the average core diameter.

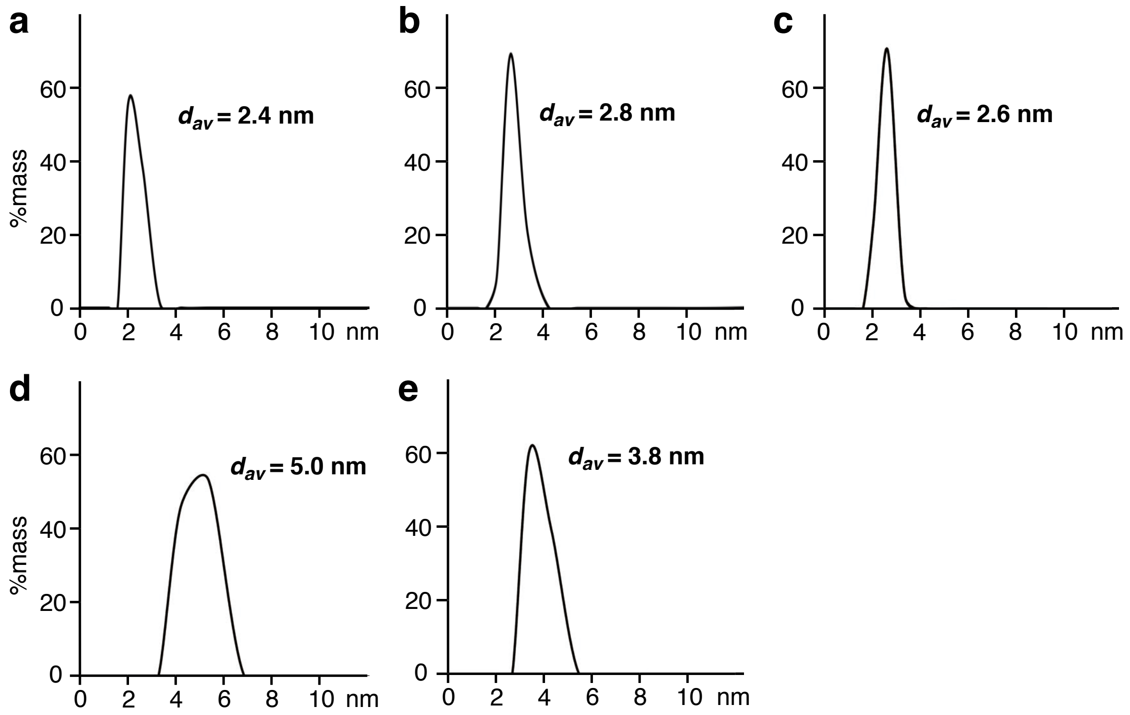

Supplementary Fig. 52 DLS charts (H_2O, 0.1 mM based on **o-1b**, room temperature) of (a) **2b**•$(\mathbf{NR})_m$, (b) **2b**•$(\mathbf{CP})_m$, (c) **2b**•$(\mathbf{C_{60}})_m$, (d) **2b**•$(\mathbf{SP})_m$, and (e) **2b**•$(\mathbf{ZT})_m$.

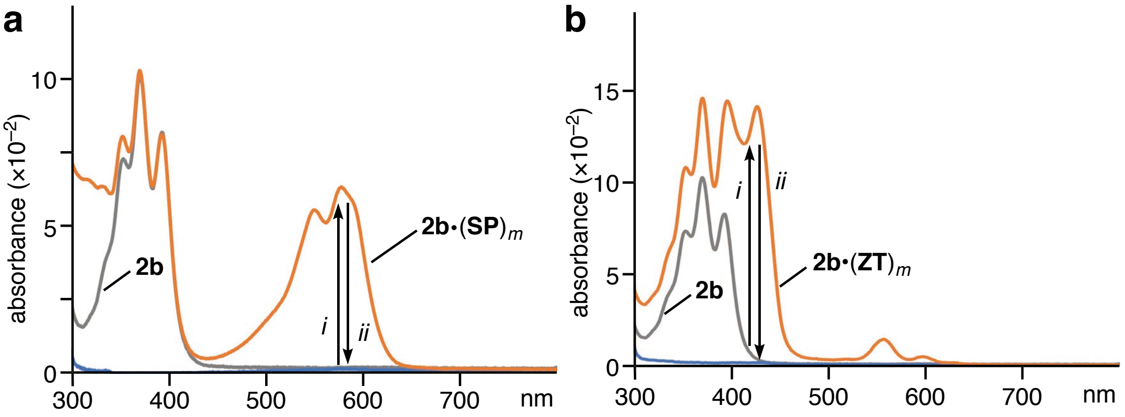

Supplementary Fig. 53 UV-vis spectra (H_2O, 0.1 mM based on **o-1b**, room temperature) of (a) (*i*) **2b** after encapsulation of **SP** and (*ii*) the product from **2b•(SP)**$_m$ after 19 h upon UV-light irradiation (λ = 380 nm, 10 min) and (b) (*i*) **2b** after encapsulation of **ZT** and (*ii*) the product from **2b•(ZT)**$_m$ after 5 h upon UV-light irradiation (λ = 380 nm, 10 min).

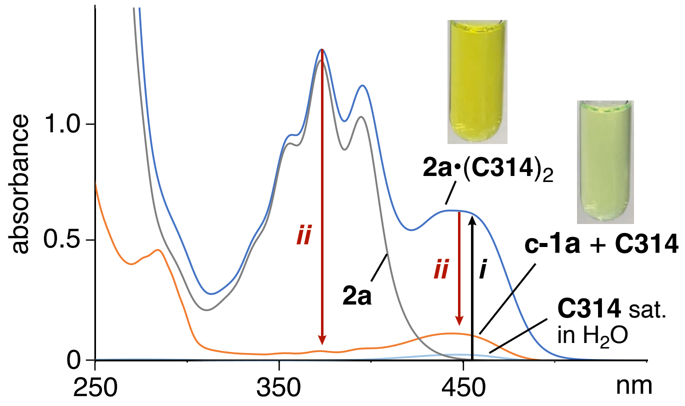

Supplementary Fig. 54 UV-visible spectra (H_2O, room temperature, 1.0 mM based on **o-1a**) of **2a** (*i*) after **C314** uptake and (*ii*) subsequent light irradiation of the product at 380 nm for 6.5 min and their photographs.

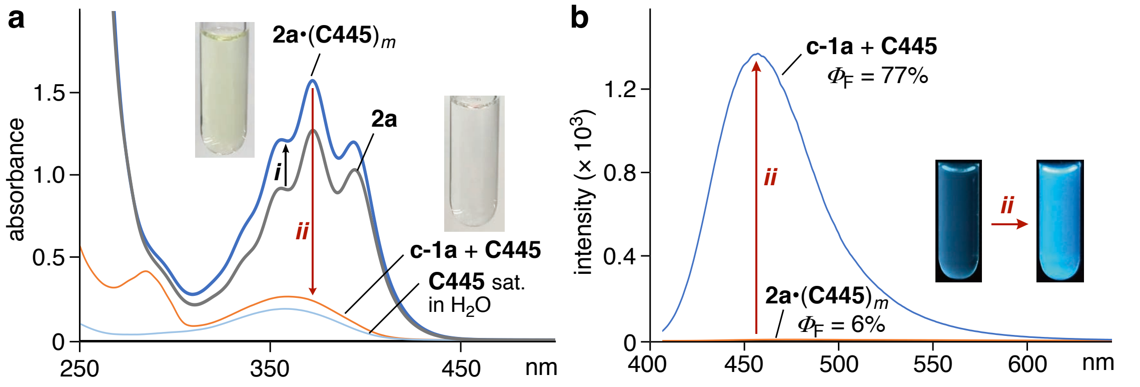

Supplementary Fig. 55 a) UV-visible spectra (H_2O, room temperature, 1.0 mM based on **o-1a**) of **2a** (*i*) after **C445** uptake and (*ii*) subsequent light irradiation of the product at 380 nm for 6.5 min (with their photographs), and b) their fluorescence spectra (room temperature, λ_{ex} = 397 nm), fluorescence quantum yields, and photographs (λ_{ex} = 365 nm).

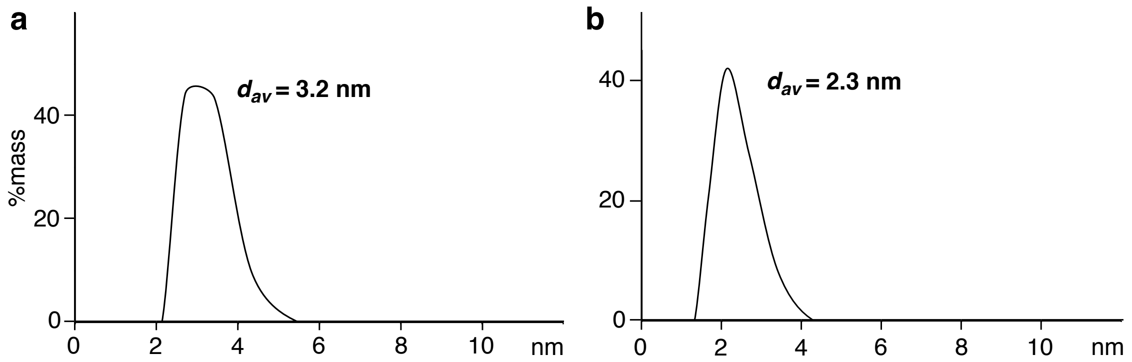

Supplementary Fig. 56 DLS charts (H_2O, 1.0 mM based on **o-1a**, room temperature) of (a) **2a•(C314)**$_m$ and (b) **2a•(C445)**$_m$.

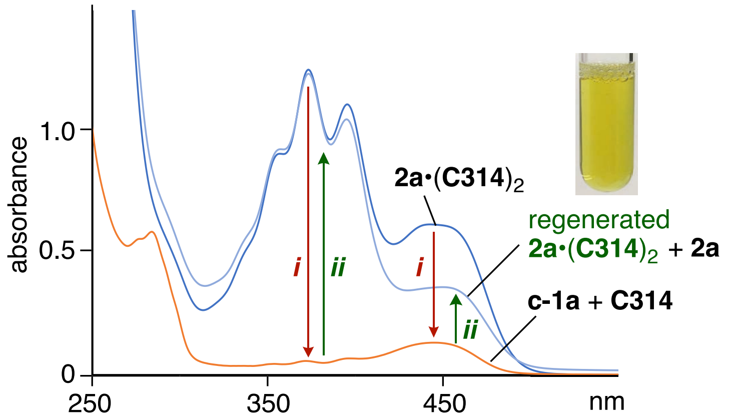

Supplementary Fig. 57 UV-visible spectra (H_2O, room temperature, 1.0 mM based on **o-1a**) of **2a•(C314)**$_2$ (*i*) after irradiation at 380 nm for 6.5 min under N_2 and (*ii*) subsequent heating at 160 °C for 30 min, addition of new **C314** and sonication for 30 min (35 kHz, 100 W), including a photograph of the solution.

Supplementary Methods

Concentration-dependent assembly of o-1a

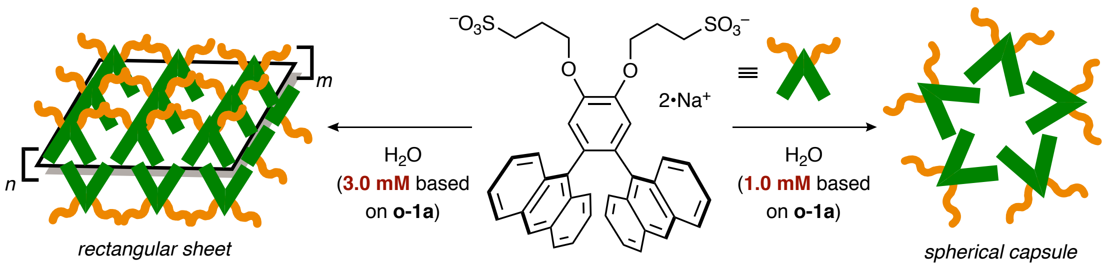

Compound **o-1a** (4.45 mg, 5.93 μmol) and H_2O (1.98 ml) were added to a glass test tube. After sonication (35 kHz, 100 W) for 15 min, the solution was filtered using a membrane filter (pore size: 200 nm). The resulting assembly (3.0 mM based on **o-1a**) was subsequently studied using UV-vis, fluorescence, and AFM analyses. In addition, concentration-dependent UV-vis and fluorescence analyses were performed by stepwise dilution of the 3.0 mM sample.

Stability of nanocapsule 2a against organic solvent and heat

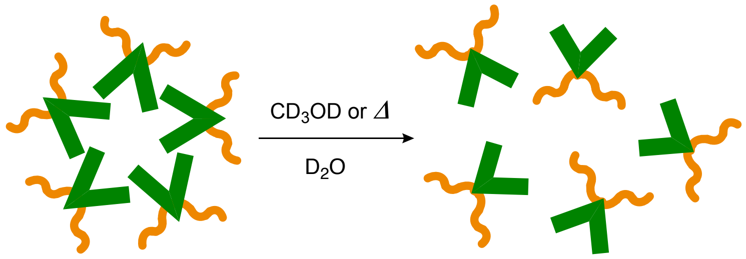

Stability against methanol: A D_2O (2.5 ml) stock solution of nanocapsule **2a** (3.76 mg, 5.00 μmol based on **o-1a**) was prepared and diluted with D_2O and CD_3OD to give a series of D_2O/CD_3OD solutions (0.6 ml each) of **o-1a** (0.45 mg, 0.60 μmol; 1.0 mM) with 10, 20, 30, and 40% CD_3OD by volume. The solutions were subsequently analyzed via ^1H NMR spectroscopy using a capillary with TMS in $CDCl_3$ as a calibration standard. The point of the complete disassembly of **2a** was defined as the point, at which the anthracene protons H_a of **2a** show a similar chemical shift towards the residual protons as those of **o-1a** in pure CD_3OD.

Stability against heat: A D_2O solution (0.4 ml) of nanocapsule **2a** (0.30 mg, 0.40 μmol based on **o-1a**; 1.0 mM) containing 1 μl DMSO as an internal standard was transferred into a high pressure NMR tube (N-5HP, Nihon Seimitsu Kagaku Co. Ltd.). The solution

was subsequently analyzed via variable temperature ^{1}H NMR spectroscopy until the point of the complete disassembly of **2a**. The disassembly temperature was defined as the point, at which the anthracene protons H_a of **2a** show a similar chemical shift towards the residual protons as those of **o-1a** in pure CD_3OD.

Photoreactivity of nanocapsule 2a in water

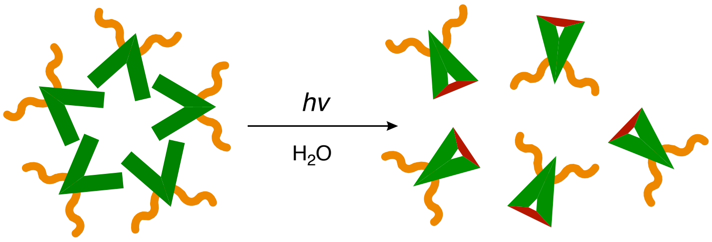

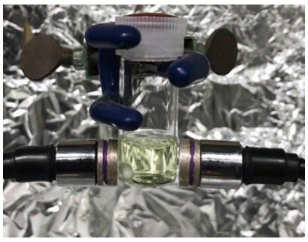

A D_2O solution (0.5 ml) of nanocapsule **2a** (0.38 mg, 0.50 µmol (1.0 mM) based on **o-1a**) was irradiated with 380 nm UV light (3 W × 2; see the photograph) for 10 min at room temperature. The ^{1}H NMR spectrum of the resultant solution showed the complete disappearance of the anthracene peaks from **2a** and the appearance of new peaks for **c-1a**. The successful photocyclization was furthermore evidenced by the UV-vis spectrum. Disassembly of **2a** via irradiation at 380 nm was confirmed via DLS and DOSY NMR analyses. In contrast, amphiphile **o-1a** is stable in the solid state and can be handled under light. Even after irradiation of solid **o-1a** for 10 min at 380 nm, no formation of **c-1o** could be observed in the ^{1}H NMR after dissolution in DMSO-d_6.

General remark: The solutions of nanocapsule **2** should always be handled under exclusion of light, since especially sunlight can lead to the formation of **c-1**.

Monitoring of the disassembly process of nanocapsule 2a in water

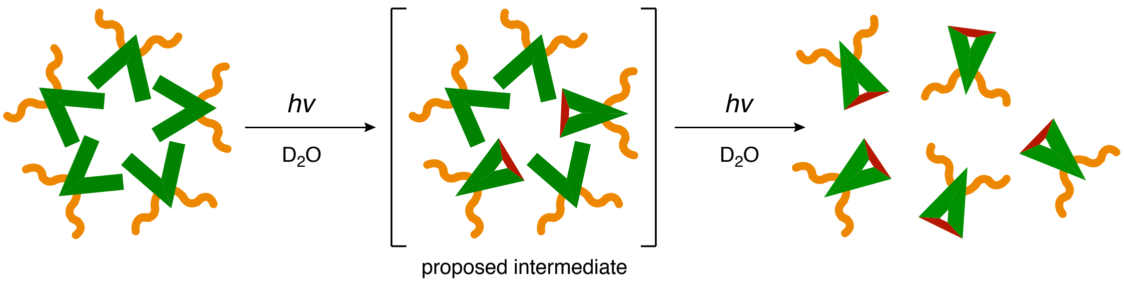

A D_2O solution of nanocapsule **2a** (1.0 mM based on **o-1a**) was irradiated in an NMR tube in steps with 380 nm UV light (3 W × 2) at room temperature. After each

step, a ^{1}H NMR spectrum was measured using a capillary with TMS in $CDCl_3$ as a calibration standard. Complete conversion from **2a** to **c-1a** was observed after 120 sec. The ^{1}H NMR spectrum after 20 sec showed a new, broad downfield-shifted signal, which suggests the formation of intermediary hetero-assemblies $(\mathbf{o\text{-}1a})_n\bullet(\mathbf{c\text{-}1a})_m$. The spectrum indicates an estimated conversion of 50% and does not correspond to a diluted ^{1}H NMR spectrum of **2a** (0.5 mM based on **o-1a**), which excludes a mere concentration effect.

Regeneration of nanocapsule 2a from c-1a via irradiation

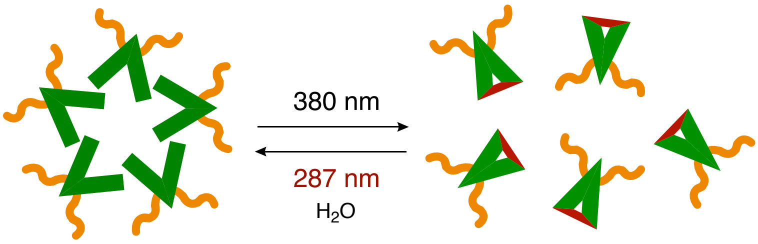

The partial regeneration of nanocapsule **2a** from closed amphiphile **c-1a** in water was established using UV irradiation at 287 nm (optimized wavelength). In an amber glass vial were added amphiphile **o-1a** (0.90 mg, 1.20 μmol) and H_2O (1.2 ml). After 2 min of sonication (35 kHz, 100 W), the solution was filtered using a membrane filter (pore size: 200 nm). An aliquot (0.4 ml) of this solution was transferred into a 1.0 mm UV-vis cell and a reference UV-visible spectrum of **2a** (1.0 mM) was measured. The cell was subsequently irradiated at 380 nm for 5 min and complete conversion into **c-1a** was then confirmed via UV-visible analysis. Next, the cell was irradiated at 287 nm (950 V, 20 nm excitation slit) for 25 min under air using a Hitachi F-7000 fluorescence spectrophotometer. The UV-visible analysis indicated successful regeneration of **2a** in 77% yield (based on the intensity of the anthracene absorption at 373 nm). The process of closing at 380 nm and reopening at 287 nm was repeated for four more times, without any significant decomposition (~4%). The clean conversion of **c-1a** to **o-1a** via irradiation was also confirmed by NMR analysis. Irradiation of a 1.0 mM solution (0.5 ml) of **c-1a** in DMSO-d_6 at 287 nm for 25 min gave a mixture of **c-1a** and **o-1a** (39% recovery), as confirmed by the ^{1}H NMR analysis.

Regeneration of nanocapsule 2a from c-1a via heating

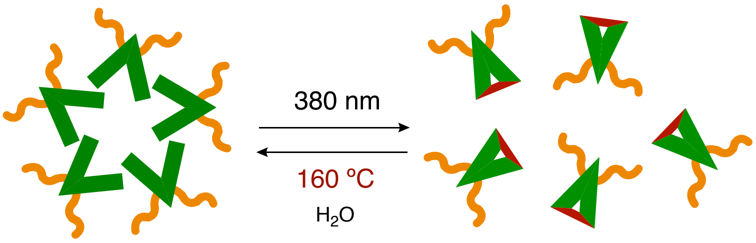

The quantitative regeneration of nanocapsule **2a** from closed amphiphile **c-1a** in water was accomplished at 160 °C in a microwave reactor. In an amber glass vial were added amphiphile **o-1a** (2.17 mg, 2.89 μmol) and H_2O (2.9 ml). After 3 min of sonication (35 kHz, 100 W), the solution was filtered using a membrane filter (pore size: 200 nm) and a UV-vis spectrum was measured as a reference for **2a** (1.0 mM). An aliquot (1.6 ml) of this solution was then transferred into a microwave tube (tube size: 0.5-2.0 ml) and subsequently irradiated at 380 nm for 5 min under stirring, yielding **c-1a** in a quantitative fashion according to UV-vis analysis. After sealing the tube, the colorless solution was stirred for 30 min at 160 ℃ in a microwave reactor (Biotage Initiator+). The now yellow solution was allowed to cool to room temperature over 15 min and a UV-vis spectrum was measured that demonstrated complete recovery of **2a** from reopened amphiphile **o-1a**. The UV-vis sample was transferred back into the microwave tube and the process of closing at 380 nm and reopening at 160 °C was repeated for four more times. After the 5th regeneration of **2a** via microwave heating, the aqueous solution was lyophilized over night. The ^{1}H NMR analysis of the resulting solid in DMSO-d_6 revealed no decomposition of **o-1a**.

Regeneration of nanocapsule 2b from c-1b via heating

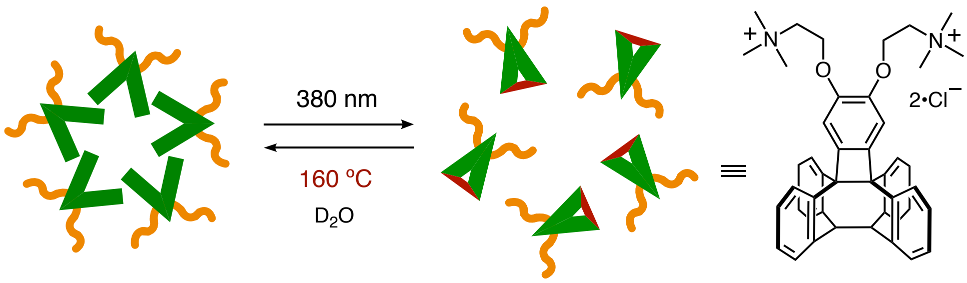

In a manner similar to **2a**, a D_2O solution of nanocapsule **2b** (0.1 mM based on **o-1b**) was irradiated with a LED lamp (λ = 380 nm, 3 W × 2) for 10 min to afford **c-1b**

quantitatively. Upon heating at 160 ºC for 30 min in a microwave reactor, nanocapsule **2b** was quantitatively regenerated in water, which was monitored by ^{1}H NMR analysis.

Encapsulation and release of CP by nanocapsule 2b

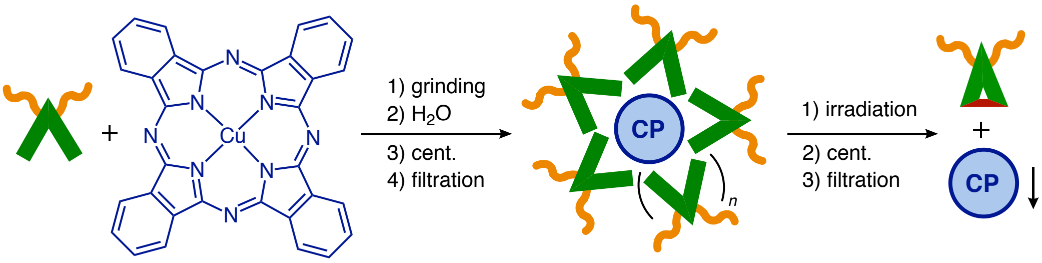

By the same procedure as **2b•**(**NR**)$_m$, host-guest composite **2b•**(**CP**)$_m$ was prepared using **o-1b** (0.30 mg, 0.43 μmol), Cu(II)-phthalocyanine (**CP**: 0.12 mg, 0.21 μmol) and H_2O (4.3 ml). The encapsulation of (**CP**)$_m$ by **2b** and the product diameter were confirmed by UV-vis and DLS analyses in water. The observed, board absorption bands for **CP** and the DLS peak for the product suggest that stacked (**CP**)$_m$ molecules (m = 2-3) are encapsulated by **2b**. The host-guest ratio (**o-1b**:**CP** = 11:1) was estimated by UV-vis analysis in 1-chloronaphthalene after the lyophilization of the product. Photoresponsive quantitative release of **CP** was also observed in the H_2O solution of **2b•**(**CP**)$_m$ by the same way. After 2 h, the suspended **CP** aggregates were fully separated by centrifugation and filtration.

Encapsulation and release of C_{60} by nanocapsule 2b

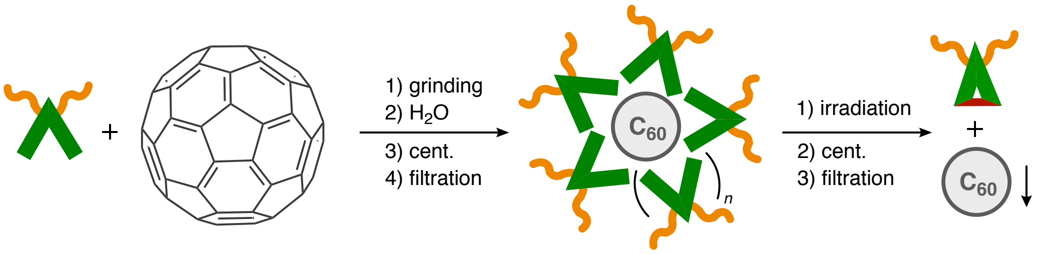

By the same procedure as **2b•**(**NR**)$_m$, host-guest composite **2b•**($\mathbf{C_{60}}$)$_m$ was prepared using **o-1b** (0.34 mg, 0.48 μmol), fullerene C_{60} ($\mathbf{C_{60}}$: 0.17 mg, 0.24 μmol) and H_2O (4.8 ml). The encapsulation of ($\mathbf{C_{60}}$)$_m$ by **2b** and the product diameter were confirmed by UV-vis and DLS analyses in water. The host-guest ratio (**o-1b**:$\mathbf{C_{60}}$ = 6:1) was estimated by UV-vis analysis in toluene after the lyophilization of the product. Photoresponsive quantitative release of $\mathbf{C_{60}}$ was also observed in the H_2O solution of

2b•(C_{60})$_m$ by the same way. After 48 h, the suspended **C_{60}** aggregates were fully separated by centrifugation and filtration.

Encapsulation and release of SP by nanocapsule 2b

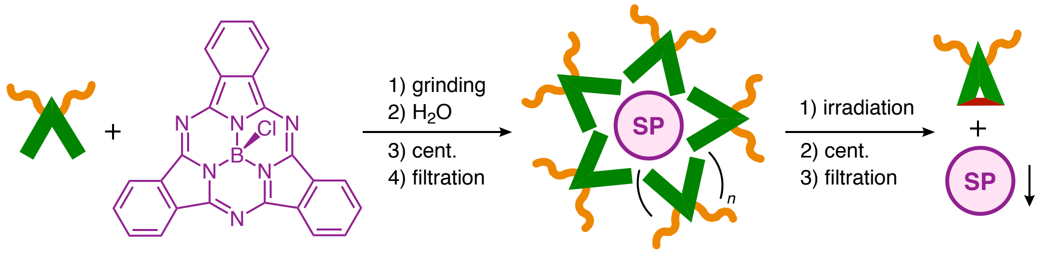

By the same procedure as **2b•(NR)**$_m$, host-guest composite **2b•(SP)**$_m$ was prepared using **o-1b** (0.31 mg, 0.44 μmol), subphthalocyanine (**SP**: 0.10 mg, 0.23 μmol) and H_2O (4.4 ml). The encapsulation of (**SP**)$_m$ by **2b** and the product diameter were confirmed by UV-vis and DLS analyses in water. The host-guest ratio (**o-1b**:**SP** = 4:1) was estimated by UV-vis analysis in $CHCl_3$ after the lyophilization of the product. Photoresponsive quantitative release of **SP** was also observed in the H_2O solution of **2b•(SP)**$_m$ by the same way. After 19 h, the suspended **SP** aggregates were fully separated by centrifugation and filtration.

Encapsulation and release of ZT by nanocapsule 2b

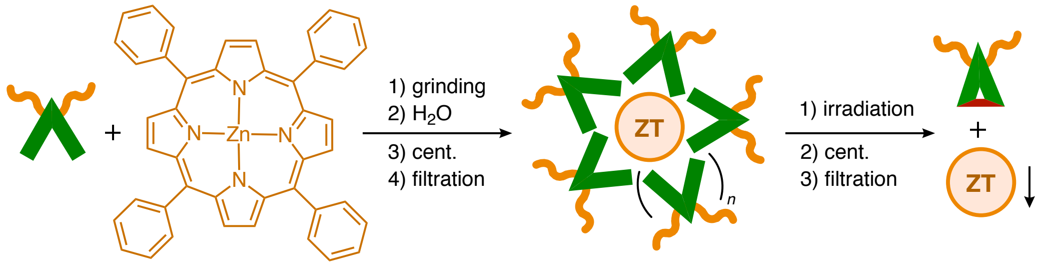

By the same procedure as **2b•(NR)**$_m$, host-guest composite **2b•(ZT)**$_m$ was prepared using **o-1b** (0.31 mg, 0.44 μmol), Zn(II)-tetraphenylporphyrin (**ZT**: 0.14 mg, 0.21 μmol) and H_2O (4.4 ml). The encapsulation of (**ZT**)$_m$ by **2b** and the product diameter were confirmed by UV-vis and DLS analyses in water. The host-guest ratio (**o-1b**:**ZT** = 8:1) was estimated by UV-vis analysis in $CHCl_3$ after the lyophilization of the product. Photoresponsive quantitative release of **ZT** was also observed in the H_2O solution of **2b•(ZT)**$_m$ by the same way. After 5 h, the suspended **ZT** aggregates were fully separated by centrifugation and filtration.

Fluorescence switching of coumarin dyes

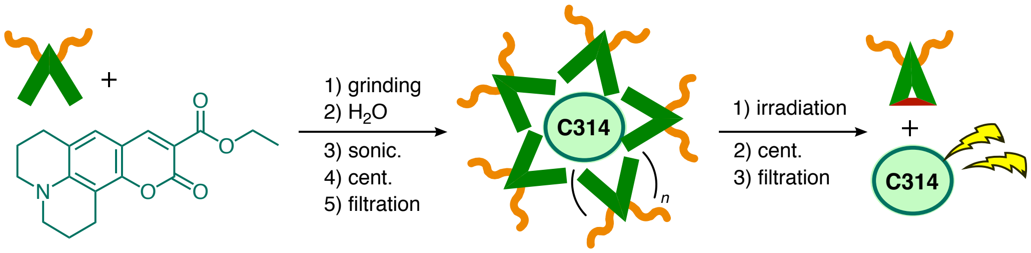

A mixture of amphiphile **o-1a** (6.03 mg, 8.03 μmol) and coumarin 314 (**C314**; 0.76 mg, 2.43 μmol) was ground for 5 min using an agate mortar and pestle. After addition of H_2O (8.0 ml), the suspension was subsequently stirred for 5 min using a pestle in dark. The suspension was sonicated for 10 min (35 kHz, 100 W) in an amber glass vial. The resultant mixture was centrifuged (16000 g, 10 min) and then filtered using a membrane filter (pore size: 200 nm) to give a clear, yellow aqueous solution of **2a•(C314)**$_m$. The absorption band of **C314** around 450 nm in the UV-vis spectrum indicated efficient uptake of **C314** by **2a**. The ^{1}H NMR spectrum of the product in DMSO-d_6 suggested the formation of a (**o-1a**)$_n$•(**C314**)$_m$ (n = ~5, m = ~2) composite. After degassing of the solution under sonication for 15 min, an aliquot (4.0 ml) of the solution was taken as a sample for the encapsulated state of the fluorophore.

Next, the solution was stirred for 6.5 min at 380 nm irradiation (3 W × 2) under N_2 to yield a turbid yellow suspension. After 2 h, the suspension was centrifuged (16000 g, 10 min) and then filtered by a membrane filter (pore size: 200 nm) to give a clear, yellow solution containing **c-1a** and released **C314** as small aggregates. Quantum yields of **2a** and **c-1a** in H_2O (λ_{ex} = 445 nm, 1.0 mM based on the monomer) were determined to be 0.8 and 1.9%, respectively.

Uptake and release of **C445** was performed by the same way. Due to the increased water-solubility of **C445**, the examined amphiphile **o-1a** to guest ratio was lowered to 1:0.14. Centrifugation and filtration were performed after 1 h following irradiation at 380 nm. The host-guest ratio of (**o-1a**)$_n$•(**C445**)$_m$ (n = ~5, m = ~2) was estimated by the ^{1}H NMR spectrum after lyophilization of the product and dissolution in DMSO-d_6. The disassembly of (**o-1a**)$_n$•(**C445**)$_m$ was, like in the case of **C$_{60}$**, not significantly influenced by the guest absorption at 380 nm, which can be explained by the almost complete coverage of the guest by the anthracene panels of **2a**.

Re-uptake of C314 by regenerated nanocapsule 2a from c-1a

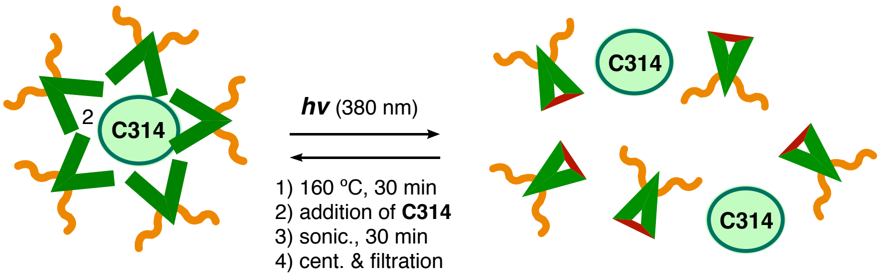

According to the procedure described above, a suspension containing closed amphiphile **c-1a** and released **C314** was produced via irradiation of **2a•**(**C314**)$_2$ at 380 nm for 6.5 min under N_2. After 2 h, the suspension was transferred into a microwave tube and subsequently stirred at 160 ºC for 30 min in a microwave reactor. The mixture was allowed to cool to room temperature over 1 h and complete re-opening of the amphiphile was confirmed via UV-vis analysis. Due to decomposition of **C314** under the microwave conditions, new **C314** (0.5 equiv. based on **o-1a**) was added and the suspension was vigorously stirred for 1 h at room temperature, followed by 30 min of sonication (35 kHz, 100 W). Centrifugation (16000 g, 10 min) and filtration with a membrane filter (pore size: 200 nm) yielded a clear, yellow solution containing **2a•**(**C314**)$_2$ (59% regeneration, based on the intensity of the guest absorption at 450 nm).
